# Metabolic versatility of small archaea Micrarchaeota and Parvarchaeota

**DOI:** 10.1038/s41396-017-0002-z

**Published:** 2017-12-08

**Authors:** Lin-Xing Chen, Celia Méndez-García, Nina Dombrowski, Luis E. Servín-Garcidueñas, Emiley A. Eloe-Fadrosh, Bao-Zhu Fang, Zhen-Hao Luo, Sha Tan, Xiao-Yang Zhi, Zheng-Shuang Hua, Esperanza Martinez-Romero, Tanja Woyke, Li-Nan Huang, Jesús Sánchez, Ana Isabel Peláez, Manuel Ferrer, Brett J. Baker, Wen-Sheng Shu

**Affiliations:** 1 State Key Laboratory of Biocontrol, Guangdong Key Laboratory of Plant Resources, College of Ecology and Evolution, Sun Yat-Sen University, Guangzhou, 510275 People’s Republic of China; 2 Departamento de Biología Funcional-IUBA, Universidad de Oviedo, Oviedo, Spain; 3 Carl R. Woese Institute for Genomic Biology, University of Illinois at Urbana-Champaign, Urbana, USA; 4 Department of Marine Science, University of Texas Austin, Marine Science Institute, Port Aransas, TX 78373 USA; 5 Laboratory of Microbiomics, National School of Higher Studies Morelia, National University of Mexico, Morelia, Michoacan 58190 Mexico; 6 Department of Energy Joint Genome Institute, Walnut Creek, CA 94598 USA; 7 Yunnan Institute of Microbiology, Yunnan University, Kunming, 650091 People’s Republic of China; 8 Department of Ecological Genomics, Center for Genomic Sciences, National University of Mexico, Cuernavaca, Morelos, 62210 Mexico; 9 Institute of Catalysis, Consejo Superior de Investigaciones Científicas (CSIC), Madrid, Spain; 10 School of Life Sciences, South China Normal University, Guangzhou, 510631 People’s Republic of China

## Abstract

Small acidophilic archaea belonging to Micrarchaeota and Parvarchaeota phyla are known to physically interact with some Thermoplasmatales members in nature. However, due to a lack of cultivation and limited genomes on hand, their biodiversity, metabolisms, and physiologies remain largely unresolved. Here, we obtained 39 genomes from acid mine drainage (AMD) and hot spring environments around the world. 16S rRNA gene based analyses revealed that Parvarchaeota were only detected in AMD and hot spring habitats, while Micrarchaeota were also detected in others including soil, peat, hypersaline mat, and freshwater, suggesting a considerable higher diversity and broader than expected habitat distribution for this phylum. Despite their small genomes (0.64–1.08 Mb), these archaea may contribute to carbon and nitrogen cycling by degrading multiple saccharides and proteins, and produce ATP via aerobic respiration and fermentation. Additionally, we identified several syntenic genes with homology to those involved in iron oxidation in six Parvarchaeota genomes, suggesting their potential role in iron cycling. However, both phyla lack biosynthetic pathways for amino acids and nucleotides, suggesting that they likely scavenge these biomolecules from the environment and/or other community members. Moreover, low-oxygen enrichments in laboratory confirmed our speculation that both phyla are microaerobic/anaerobic, based on several specific genes identified in them. Furthermore, phylogenetic analyses provide insights into the close evolutionary history of energy related functionalities between both phyla with Thermoplasmatales. These results expand our understanding of these elusive archaea by revealing their involvement in carbon, nitrogen, and iron cycling, and suggest their potential interactions with Thermoplasmatales on genomic scale.

## Introduction

Archaea constitute a considerable portion of microbial diversity, and play significant roles in many biogeochemical cycles on Earth [1]. However, compared to bacteria they are much less understood and fewer genomes have been sequenced [2]. The recently delineated superphylum DPANN includes several phyla of archaea with small cell and genome sizes and limited metabolic capabilities [3–6]. To date, 48 DPANN draft genomes are available (Supplementary Table 1; see references therein) and only two symbiotic Nanoarchaeota co-cultures have been obtained [5, 7].

Two DPANN phyla, Micrarchaeota and Parvarchaeota, referred to as Archaeal Richmond Mine Acidophilic Nanoorganisms (ARMAN), were first reported in acid mine drainage (AMD) biofilms of Iron mountain (Richmond, CA, USA) and are among the smallest microorganisms described to date [8, 9]. The AMD biofilms in Iron Mountain have been comprehensively studied for microbial ecology and evolution [10]. Four genomes of ARMAN have been obtained from this site, including ARMAN-1 and ARMAN-2 from Micrarchaeota, and ARMAN-4 and ARMAN-5 from Parvarchaeota. The metabolic functions of ARMAN-2, -4 and -5 in the AMD biofilms have been speculated based on metaproteomic analyses [11], while ARMAN-1 was published recently only to report its CRISPR–Cas system [12]. Interestingly, ARMAN cells were observed having interactions with Thermoplasmatales cells via pili-like structures [11], and this phenomenon was further documented using cryogenic transmission electron microscope technology [13], while the ecological significance of such interactions remains unclear. Moreover, ARMAN-specific PCR primers and metagenomics have revealed their occurrence in many other AMD-related environments [14–17], indicating wide distributions of related microorganisms in nature.

Despite these investigations described above, we know little about their biodiversity, environmental distribution (in other acidic and non-acidic environments), physiologies, and roles in biogeochemical cycling. To address these gaps, we assembled and binned 39 new genomes from metagenomic datasets obtained from two AMD and three hot spring related environments around the world, and the environmental distribution of Micrarchaeota and Parvarchaeota taxa were evaluated by analyzing 16S rRNA gene sequences from those new genomes and NCBI and IMG/M databases. Metabolic potentials of Micrarchaeota and Parvarchaeota were predicted based on functional annotation of their genes, to reveal their metabolic functions and potential roles in nature. Additionally, genomic information likely suggested that ARMAN spp. are microaerobic and/or anaerobic, thus enrichments with inoculum from AMD systems were performed for these elusive archaea (Supplementary Fig. 1).

## Materials and methods

### ARMAN genomes and related metagenomes in public database

In NCBI database, there are two Micrarchaeota and two Parvarchaeota genomes reported from Iron Mountain: ARMAN-1 (NCBI accession number, PRJNA349044), ARMAN-2 (PRJNA38565), ARMAN-4 (PRJNA38567), and ARMAN-5 (PRJNA38569), which were included for analyses in this study.

A metagenomic data set sampled from the Fankou mine tailings AMD outflow in 2010 (sample abbreviation: FK_AMD_2010), which was published previously [15], was included in this study to retrieve ARMAN genomes from newly assembled contigs. The ultra-small cells were collected by filtering the AMD outflow through 0.22 μm pore size filters, which were preserved for DNA extraction [15].

The available ARMAN genomes were also used as references to search for additional ARMAN sequences in publicly available contigs/scaffolds of >3000 metagenomic data sets deposited in the IMG/M system (alignment length ≥ 250 bp, similarity ≥ 75%). This analysis retrieved one additional metagenomic data set from Obsidian Pool in the Yellowstone National Park (SRA accession, SRP099390; IMG genome ID, 3300002966) with ARMAN-related genomic sequences (sample abbreviation: YNP_OP). Obsidian Pool (N44.36°, W110.26°) was reported as a mesothermal hot spring (56 °C; Supplementary Table 2) [18].

### Study sites, sampling, DNA extraction, and sequencing

Metagenomic data sets from two AMD and two hot spring environments were collected and analyzed in this study (Supplementary Fig. 2 and Supplementary Table 2); the site descriptions and experimental procedures (e.g., sampling, DNA extraction and sequencing) are detailed below.

1. Fankou AMD outflow (FK_AMD_2014). Fankou mine is located in north Guangdong province of China (N25.05°, E113.67°), and the microbial ecology of this site was deeply studied previously, to reveal the microbial diversity and composition at spatial and temporal scales of the mine tailings [19], the shifts of microbial composition and function across the tailings acidification processes [20], and also the metatranscriptomic activities in the related AMD systems [14, 15]. Microorganisms in AMD outflow were collected in September, 2014, using filters with different pore sizes (i.e., 1.6, 0.45, and 0.22 μm). A total of 20 L AMD outflow was successively filtered through the abovementioned three filters, then the 0.22 μm filters with ultra-small cells on them were packaged in 50 mL sterile tubes and preserved in liquid nitrogen when transporting to the lab, and stored at −80 °C until DNA extraction within 24 h. Community genomic DNA was extracted from the filters as described previously [14]. The total genomic DNA was sequenced using Illumina paired-end (PE) 125 and 250 bp kits on a HiSeq2500 platform with an insert length of ~400 bp. It should be pointed out that, this sampling approach (also used for FK_AMD_2010) may miss some smaller members of the population, for some microbial community members could pass through 0.22 μm pore size filters [8, 21].
2. Fankou AMD sediment (FK_Sedi). A core was sampled from the Fankou AMD pond using a sediment-sampling collector in March, 2015. The sediment core was divided into five layers based on their different colors, which generally reflect their oxidative status. Each of the five layers were collected in 50 mL sterile tubes, stored in an icebox and transported to the lab within 4 h after sampling, and stored at 4 °C until DNA extraction within 24 h. DNA of the AMD sediment was extracted using the FastDNA SPIN kit (MP Biomedicals, Irvine, CA, USA) according the manufacturer’s instructions. The extracted DNA was individually amplified using multiple displacement amplification (MDA) with the GenomiPhi DNA amplification kit (v3) (Amersham Biosciences, Piscataway, NJ), and purified for library construction and sequenced on an Illumina HiSeq2500 platform with PE 150 bp kits.
3. Los Rueldos (LR_AMD). This sampling site is located in the Morgao stream, 2 km northeast of the town of Mieres and 20 km southeast of Oviedo, the capital city of Asturias, NW Spain (N43.27°, W5.77°). Previous analyses have revealed the existence of ARMAN taxa in the AMD of this site [16]. Two macroscopic streamer samples (one oxic and one anoxic) thriving along an AMD outflow were collected, and filtered with 0.45 μm filters. The genomic DNA was extracted from the filtration within 2 h as previously described [16], and amplified using the GenomiPhi DNA amplification kit (v3) (Amersham Biosciences, Piscataway, NJ). The DNA samples were used for library construction, and sequenced on an Illumina HiSeq2000 platform with a PE 100 bp kit.
4. Tengchong geothermal area (TC_Endo). The Tengchong geothermal area is located in the south of Yunnan province of China (N24.95°, E98.44°), representing the largest geothermal area in China. Endolithic communities were detected in the surface of a rock near a hot spring fumarole (referred to as the sampling site of “Drty” in [22]). Endolithic community samples were collected with a sterile iron spoon (~0.5 cm from the outside) in December, 2014, and stored in an icebox when transporting to the lab within 24 h, and stored at 4 °C until DNA extraction within 24 h. DNA extraction methods were the same as used for AMD outflow filters, except that no liquid nitrogen was used for breaking the cells. The total genomic DNA was sequenced using Illumina paired-end (PE) 125 and 250 bp kits on a HiSeq2500 platform with an insert length of ~400 bp.
5. Los Azufres National Park (Me_Mat). The Los Azufres National Park is located in the west of Mexico, 65 km east of the city of Morelia (N19.47°, W100.39°). Microbial layer samples were collected during April 2013 at the Los Azufres geothermal field in a rocky area full of cracks from which hot fumaroles emerge, just in front of the muddy lagoon known as Los Azufres Natural Spa. DNA was extracted using the MOBIO Mega Prep Soil DNA extraction kit, and sequenced on an Illumina Miseq platform using a PE 250 bp kit.

### Metagenome assembly and genome binning

All the raw data of the metagenomic data sets were filtered to remove low quality bases/reads using Sickle software (version 1.33; [23]), with the parameters set to “-q = 15 and −l = 50” (Supplementary Table 3). Afterwards, one or more quality datasets of each sampling site were assembled (or co-assembled for the five AMD sediments, and for the two Los Rueldos AMD streamers) using SPADES (version 3.7.1; [24]) with a proper kmer set (Supplementary Table 3), under the “--meta” model. To calculate scaffold coverage, all high-quality reads were mapped to the assembled scaffolds using BBMap, with the parameters set as “minid = 0.97, local = t”. The coverage of each scaffold was determined based on the mapping results using MetaBAT [25]. For scaffolds assembled from co-assembled quality datasets, each metagenomic dataset was separately mapped to the scaffolds, and the coverage of each dataset was calculated. The scaffolds were binned using MetaBAT with default parameters, considering both tetranucleotide frequencies and coverage of the scaffolds (multiple coverages for co-assembled AMD sediments and Los Rueldos AMD streamers).

### Identification of ARMAN associated genomic bins

The bins obtained from MetaBAT were evaluated for taxonomic assignment, genome completeness and potential contamination, using the “lineage_wf” pipeline in CheckM [26]. To determine which of the bins belong to the ARMAN phylum, all bins that were assigned to the lineage of “Archaea” by CheckM were further assessed using the following procedures:

1. comparing their predicted genes (amino acid sequences, which were received from the individual scaffolds using Prodigal included in CheckM) against the NCBI-nr database using the DIAMOND software [27] under the BLASTp model.
2. comparing the scaffolds against the SILVA 16S/18S dataset using BLASTn. All genomic bins with >50% of genes having a best hit to ARMAN spp. from Iron Mountain (ARMAN-1, −2, −4, and −5; see above), and/or with a 16S rRNA gene sequence closely related to ARMAN spp. (sequence identify >75%), were considered as ARMAN genome bins for further evaluation.

### Genome bins optimization

The ARMAN genome bins were further optimized to obtain high-quality genomes as follows:

1. combination of genome bins. The automatic binning tool MetaBAT may separate a “true” genome bin into two or more smaller, separate bins. To combine these smaller ARMAN genome bins all scaffolds were visualized using ESOM [28] and bins that clustered together and shared a similar coverage range were combined and evaluated by CheckM.
2. removal of potential contaminations. Protein-coding genes from each ARMAN genome bin (also those combined ones) were determined using Prodigal included in CheckM (see above) and the acid amino sequences were compared against the NCBI-nr database using DIAMOND (“blastp” model; [27]). Each scaffold was evaluated for contamination based on the taxonomical assignment of its genes; and those scaffolds with abnormal coverage and/or GC content were deleted or moved to another ARMAN bin.
3. re-assembly of genome bins. Quality metagenomic reads were mapped to the scaffolds of each genome bin obtained from the former two processing steps described above, using BBMap with the parameters set as “minid = 0.97, local = t”. The mapped quality reads of each genome bin were assembled using SPADES (version 3.7.1) with the k-mer set the same as during the metagenomic assembly, under the “--careful” model.
4. removal of contaminations from the reassembled genome bins. This step was performed as stated above in step (2). The optimized genome bins were visualized using ESOM (Supplementary Fig. 3). After genome optimization, a set of 54 conserved archaeal single-copy genes (SCGs) [3] were retrieved to estimate genome completeness (Supplementary Table 4). Additionally, the genome bins were assessed using CheckM (Packs et al. 2015) to estimate the degree of contamination (Supplementary Table 5).

### Genome bins analyses

The optimized genome bins were submitted to the IMG/MER system for gene calling and annotation (Supplementary Table 5). Protein-coding genes were also assigned to functional orthologs of the KEGG Orthology database using KAAS [29] (parameters: GHOSTX, Genes dataset, SBH assignment method). Carbohydrate-active enzymes were identified using the carbohydrate-active enZYmes (CAZy) database on the dbCAN webserver [30]. A subset of CAZy genes, which were shown to be involved in specific carbohydrate degradation pathways [31], was selected for further analysis. Peptidases were identified using MEROPS [32]. All the genome bins were also uploaded to RAST (Rapid Annotation using Subsystem Technology) [33, 34] for gene prediction and annotation, using the Network-Based SEED API using the command svr_submit_RAST_job, selecting the domain Archaea and the RAST gene caller method. Metabolic pathways were constructed for Micrarchaeota and Parvarchaeota based on gene annotation results. The structural prediction of proteins was performed by the online tool PRED-SIGNAL with default parameters [35].

### KEGG Orthology based cluster analyses of ARMAN genomes

To have a global comparison of metabolisms of the ARMAN genomes, the occurrence of KEGG Orthology (KO) in each Micrarchaeota and Parvarchaeota genome were summarized based on KAAS annotation results (see above) (Supplementary Table 6), then the data was transferred to (0,1) data (for a given KO in a given genome, if the count number is 1 or more, then set as 1, if the count number is 0, then set as 0). Hierarchical clustering analysis of all the Micrarchaeota and Parvarchaeota genomes was respectively performed based on the (0,1) data, with correlation distance and complete-linkage method using the R package of “pheatmap” (version, 0.7.7).

### 16S rRNA gene sequences retrieval

ARMAN-related 16S rRNA gene sequences were retrieved from NCBI GenBank (Supplementary Fig. 1). The 16S rRNA gene sequences of ARMAN-1 and ARMAN-2 (Micrarchaeota) and ARMAN-4 (Parvarchaeota), were used as query to search for related 16S rRNA gene sequences in GenBank using BLASTn. Hits with an alignment coverage ≥50% and sequence similarity ≥75% (phylum level threshold as determined using 16S rRNA gene sequences; [36]) were downloaded from the GenBank datasets. Additionally, 21 Micrarchaeota 16S rRNA gene sequences were obtained from the IMG/M system, which were also included in this study [37]. For phylogeny analyses of these retrieved 16S rRNA gene sequences with those from the obtained genome bins (see below for methods), an initial tree was constructed to remove those sequences not related to ARMAN, as determined by the phylogenetic topology and sequence similarity between each other (≥75%). Finally, we obtained 176 Micrarchaeota and 114 Parvarchaeota related 16S rRNA gene sequences with a length ≥800 bp (Supplementary Table 7).

### Phylogenetic analyses

A total of 16 ribosomal protein sequences (i.e., L2, L3, L4, L5, L6, L14, L15, L16, L18, L22, L24, S3, S8, S10, S17, and S19) from the ARMAN genomes and NCBI Archaea genomes (including selected genomes from all known archaeal phyla) were individually aligned using Muscle [38], and filtered by TrimAL [39] to remove columns comprised of more than 95% gaps, then the 16 filtered ribosomal protein sequences were concatenated for each genome. All the concatenated sequences represented at least 50% of the expected alignment columns (29 out of 33 genomes spanned >80% of the expected alignment columns). The concatenated sequences were used to reconstruct a phylogenetic tree using RAxML version 8.1.24 [40] with the parameters set as “-f a -m PROTGAMMALG”. 16S rRNA gene sequences from all ARMAN genomes (see above; deleted insertion with a length ≥10 bp) and other archaea [2] were aligned using the SINA alignment algorithm [41] through the SILVA web interface [42], the alignment was filtered by TrimAL to remove columns comprised of more than 95% gaps and a phylogenetic tree was constructed using RAxML version 8.1.24 [40] with the parameters set as “-f a -m GTRGAMMA -n boot -c 25 -p 12345 -x 12345”. The resulting newick file with the best tree topology was uploaded to iTOL v3 [43] for visualization and formatting.

### Relative abundance and community composition analyses

The single-copy ribosomal protein S3 (rpS3) was used as a phylogenetic marker for taxonomic assignment and relative abundance analyses as previously reported [3]. For each metagenomic assembly result, all rpS3 sequences were identified by CheckM [26], and the coverage was determined based on the coverage from the corresponding scaffold (see above). The relative abundance of each taxa was calculated by dividing the coverage of its rpS3 by the total coverage of all rpS3 proteins in the community.

### Micrarchaeota and Parvarchaeota enrichment experiment

An enrichment attempt of ARMAN cells was performed under microaerobic/anaerobic conditions in laboratory, to illustrate they could grow with low O_2_ [11, 17]. We have a stimulated AMD system located in the campus of Sun Yat-sen University, which is a 1 × 1 × 1 m cement-lined pit with approximately 5 t natural pyrite particles (from Yunfu sulfur/iron mine of Guangdong, China), and purified water was injected into the pit monthly to accelerate the pyrite acidification processes, and the microbial communities dynamics have been reported previously [44].

In August of 2016, the AMD outflow samples collected from the stimulated system were filtered through pore sizes of 0.45 μm filters, and the filtrate was collected into sterile tubes and stored at 4 °C for enrichment within 24 h (the filtrate will be referred as “inoculum” hereafter). Subsequently, 50 ml inoculum was centrifuged (4 °C, 12,000 × *g*, 30 min) and the supernatant was discarded, and genomic DNA was extracted using the FastDNA SPIN kit (MP Biomedicals, Irvine, CA, USA) according the manufacturer’s instructions, and PCR analyses using ARMAN-specific primer set (ARM979F/ARM1365R; [11]) indicated the occurrence of ARMAN cells in the inoculum (data not shown).

The culture medium of the novel and extremely acidophilic, cell-wall-deficient archaeon *Cuniculiplasma divulgatum* (primarily known as G-plasma of Thermoplasmatales), which was reported to co-culture with filterable archaea with reduced genome size [45], was used for ARMAN enrichment. The culture medium was modified by adjusting the pH value to 3.0 with H_2_SO_4_, and addition of 1 g Na_2_S_2_O_3_ each 100 ml, then sterilized at 115 °C for 25 min. To each 100 ml of modified and sterilized medium, 1 g of sterilized nutriment was added, that is, (1) yeast extract, (2) beef extract and (3) peptone, additionally, a control without nutriment addition was included in this experiment. Afterwards, 1 mL of inoculum (see above) was added into 100 mL of each of the four mediums (in 250 mL culture bottle). The culture bottles were sealed and located in a dark and non-shaking incubator for growth (37 °C).

After 14 days, cells from all four mediums (~100 ml) were individually collected by centrifugation (4 °C, 10,000 × *g*, 15 min), genomic DNA was extracted from the precipitates [14]. PCR using ARMAN-specific primer set was performed to detect the ARMAN occurrence, the results showed all excepting the negative control, contained ARMAN cells (Supplementary Fig. 4). Due to the relatively low biomass of these communities, the three extracted DNA samples with ARMAN cells were amplified by MDA (GenomiPhi DNA amplification kit (v3); Amersham Biosciences, Piscataway, NJ), and sequenced for metagenomic analyses. De novo assembly (co-assembly) and genome binning was performed, and the community composition and relative abundance of taxa were analyzed based on ribosomal protein S3 sequences as described above.

Previous studies showed that MDA treatment could skew the relative abundance of taxa and compromise quantitative analysis of metagenomes [46], we thus compared the results of non-MDA and MDA metagenomes of the inoculum (see above). For the inoculum DNA sample (concentration = 89 ng/μl), 1 μl was diluted × 10 times, and 1.2 μl of the diluted DNA (~10 ng) was amplified using MDA in triplicates. The three amplified DNA samples and also the raw inoculum DNA sample (non-MDA) were sequenced for metagenomic analyses (quality control, co-assembly, coverage calculation as described above). The relative abundance of taxa in the non-MDA sample and three MDA samples were evaluated based on the rpS3 sequences as described above.

### Phylogenetic analyses of interested genes

Phylogenetic analyses were performed individually for specific genes involved in the TCA cycle and the respiratory chain. For example, for a given gene, the protein sequences in all available Micrarchaeota genomes (33 in total) and Parvarchaeota genomes (16 in total) were identified and extracted, then compared against the NCBI-nr database for their closed homologies using BLASP (e-value threshold = 1e−5). The 20 top hits were extracted and double checked for their functional annotation, those ones with correct function were retained, and aligned with those from the abovementioned genomes using Muscle with default parametres [38], and filtered by TrimAL [39] to remove columns comprised of more than 95% gaps, then a phylogenetic tree was built using RAxML version 8.1.24 [40] with the parameters set as “-f a -m PROTGAMMALG” (corresponding gene sequences from *E.coli* strains were used for outgroup). The resulting newick file with the best tree topology was uploaded to iTOL v3 [43] for visualization and formatting, all the trees were rooted between the outgroup and others.

### Accession codes

All newly constructed Micrarchaeota and Parvarchaeota genomes reported in this study have been deposited in the IMG/MER system under the accession numbers listed in Supplementary Table 5.

## Results and discussion

### New Micrarchaeota and Parvarchaeota genomes reconstructed from metagenomes

A total of 10 unpublished metagenomic datasets were obtained from two AMD (one AMD outflow and five AMD sediment metagenomes from Fankou mine tailings of China, and two AMD streamer metagenomes from Los Rueldos of Spain) and two hot spring related environments (one hot spring endolithic metagenome from Tengchong of China, one hot spring mat metagenome from Los Azufres National Park of Mexico), and another two data sets were retrieved from public databases (one AMD outflow metagenome from Fankou mine tailings of China [15]; and one hot spring sediment metagenome from Yellowstone National Park of USA) (Supplementary Figs. 1 and 2). Similar to the Iron mountain where ARMAN were first reported, all these sampling sites except the Yellowstone National Park Obsidian pool were characterized by low pH values (Supplementary Table 2).

A total of ~200 Gbp of raw sequences were obtained from all these 12 metagenomes (Materials and methods; Supplementary Table 3). Quality control, de novo assembly and subsequent genome binning from these metagenomes resulted in a total of 27 Micrarchaeota and 12 Parvarchaeota genomes (Supplementary Fig. 3). Based on the occurrence of 54 archaeal single-copy genes [3], the newly constructed genomes showed a completeness of 78–100% (93% on average), and a very low degree of genomic contamination estimates (0.8% on average) (Supplementary Table 4). These genomes have a genome size approximating 0.64–1.08 Mb, and on average encoding 912 genes for Micrarchaeota, and 897 genes for Parvarchaeota, which were comparable to those of the four published ARMAN genomes [11, 12] (Table 1 and Supplementary Table 5). Compared with the Micrarchaeota genomes (unpaired *t* test, *P* < 0.05), Parvarchaeota have significantly shorter average gene length (820 bp vs. 843 bp), higher coding density (92.2% vs. 90.4%) and higher frequency of overlapping genes (19% vs. 10%) (Table 1 and Supplementary Table 5).

**Table 1:** General features of the ARMAN genomes that newly reconstructed and previously published ARMAN genomes

	Newly reconstructed		Previously published^a^			
Micrarchaeota	Parvarchaeota		ARMAN-1	ARMAN-2^b^	ARMAN-4	ARMAN-5
No. of genomes	27	12	1	1	1	1
No. of scaffolds	3–59 (22)	7–78 (32)	66	1	44	73
Total length (Mb)	0.66–1.08 (0.85)	0.64–0.96 (0.80)	0.87	1.01	0.79	0.92
GC content (%)	28.3–54.2 (44.5)	32.9–44.5 (37.4)	45.6	48	34.4	34.2
No. of tRNA genes	27–48 (37)	30–48 (42)	36	38	43	45
No. of protein-coding genes	743–1181 (912)	701–1095 (897)	993	1069	933	1076
Gene length (bp)	793–891 (843)	767–857 (820)	793	859	767	770
Overlapped genes (%)	7–15 (10)	17–20 (19)	9	10	18	20
Coding density (%)	87.3–92.7 (90.4)	90.4–93.4 (92.2)	89.4	91.3	90.7	90.8
Estimated completeness^c^	78–100 (94)	78–98 (92)	89	100	80	85
Contamination (%)^d^	0–2.8 (0.7)	0–2.9 (1.2)	0	0.9	3.3	5.6

^a^The previously published ARMAN genomes were analyzed (annotation and others) as for newly reconstructed ones

^b^The closed genome of ARMAN-2 is reported in this study

^c^For 54 archaeal single-copy genes (SCGs) previously used for genome completeness evaluation as in [3]. Four Micrarchaeota genomes were detected with all the 54 SCGs (Supplementary Table 4), while not closed

^d^The contamination of each genome were evaluated with CheckM [26] using a set of 149 archaeal SCGs. Those in brackets are average numbers. See Supplementary Table 5 for details

### Relative abundance and microbial composition

Though 25 out of 39 new genomes were reconstructed from metagenomic data sets obtained by filtration for small cells (Materials and methods), 17 out of these 25 genomes showed a relative abundance of <1% (Supplementary Fig. 5). However, one Micrarchaeota genome (FK_Sedi_bin_12_4) was detected with a relative abundance of 5.3–21.3% in AMD sediment layers of FK_Sedi_C1_3, C1_4 and C1_5), indicating its potential significance in the corresponding AMD sediments.

Interestingly, while the microbial composition of the analyzed communities were remarkably different from each other (Supplementary Fig. 6), the “alphabet plasmas” (e.g., A-, E-, G-, I-plasma; [47]) belonging to the order Thermoplasmatales (phylum, Euryarchaeota; class, Thermoplasmata) were detected in all of them. “Alphabet plasma” are abundant inhabitants in AMD ecosystems [47, 48], our analyses indicated they may also favor acidic hot spring related environments.

### Phylogeny and biodiversity of Micrarchaeota and Parvarchaeota

**Fig. 1:**
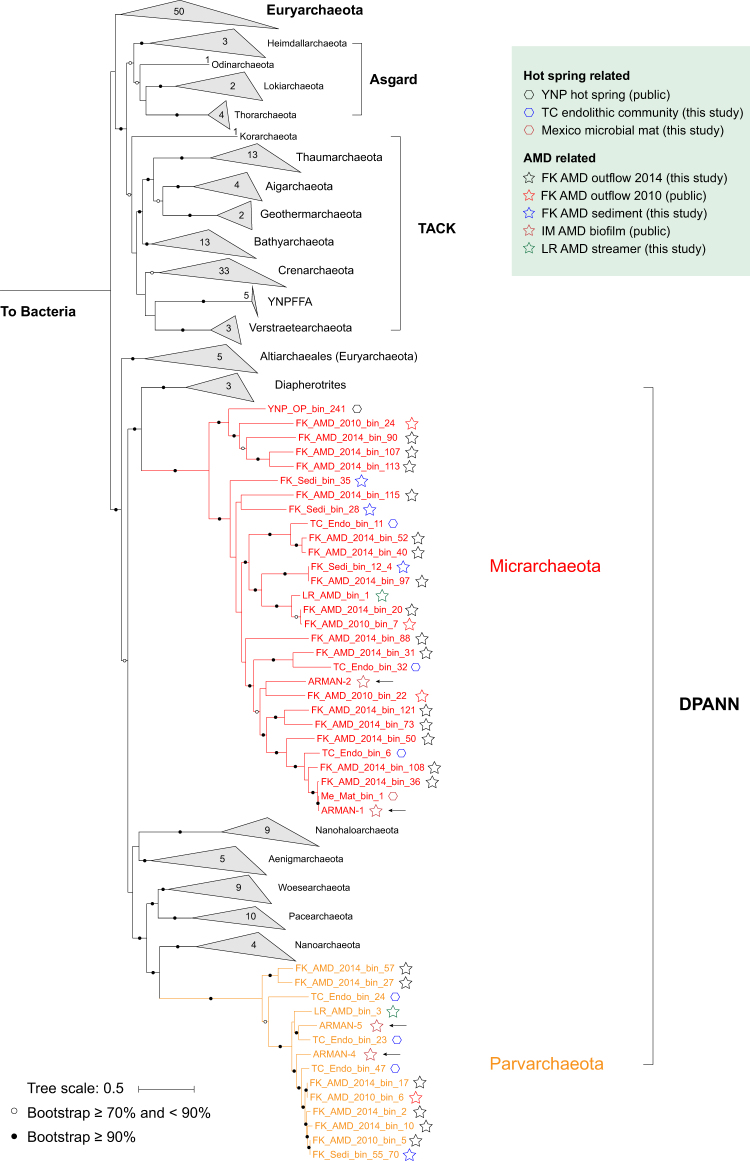
Phylogenetic analyses of Micrarchaeota and Parvarchaeota genomes The phylogenetic tree was constructed based on 16 concatenated ribosomal protein sequences from each Micrarchaeota and Parvarchaeota genome and reference archaeal genomes (or scaffolds with the target ribosomal protein sequences) (Materials and methods). The three archaeal superphyla TACK, Asgard and DPANN, and the number of genomes included for each phylum are shown. Previously published genomes are indicated by arrows, and the metagenomic datasets of “YNP hot spring” and “FK AMD outflow (2010)” were retrieved from public databases. Bootstrap values are based on 100 replicates

Based on a phylogenetic analysis of 16 concatenated ribosomal protein sequences, both Micrarchaeota and Parvarchaeota branched in the DPANN superphylum of the archaeal domain (Fig. 1), Micrarchaeota clustered with Diapherotrites, and Parvarchaeota clustered with Nanoarchaeota, Pacearchaeota, and Woesearchaeota. The 16S rRNA gene sequences based phylogeny showed a different pattern as reported in a previous study [3], with Parvarchaeota clustered with Pacearchaeota and Woesearchaeota, and then clustered with Micrarchaeota (Supplementary Fig. 7). With the availability of sufficient genomes obtained in this study, both phylogeny revealed that Micrarchaeota and Parvarchaeota are not monophyletic but two distinct phyla, and should not be combined as one [4].

Based on similarity thresholds of 16S rRNA gene sequences for taxonomic level definition (genus, 94.5%; family, 86.5%; [36]), the previously published ARMAN-1 and -2 represent two genera in a single family, as well as ARMAN-4 and -5 (Supplementary Fig. 7). In this study, the newly reconstructed Micrarchaeota genomes represented at least 12 genera within two families, and the Parvarchaeota genomes represented at least three genera within one family, greatly expanding the phylogenetic and genomic diversity of these two lesser known phyla.

### Environmental distribution of Micrarchaeota and Parvarchaeota

Comparison of Micrarchaeota 16S rRNA gene sequences from around the world revealed their considerable biodiversity, especially within AMD and hot springs. Micrarchaeota were also found in a variety of non-acidic environments, including hypersaline mats, soils, peat, freshwater lakes, and underground water, indicating their potential highly adaptive capacities and phylogenetic diversity of at least two classes (Supplementary Fig. 8). In contrast for Parvarchaeota, all the retrieved 16S sequences from NCBI GenBank were from AMD-related environments, and were affiliated within the same family as found within the Tengchong acidic endolithic community reported in this study (Materials and methods). The limited distribution of the known Parvarchaeota may be due to their lower phylogenetic diversity, indicating more Parvarchaeota clades remain to be discovered, while their low abundance in nature may hinder this process.

For those Micrarchaeota and Parvarchaeota spp. with genomic information obtained in this study, some of them showed clear environmental preference. Micrarchaeota genus 12 and Parvarchaeota genus 1 and 2 members (Supplementary Fig. 7) were all obtained from environments with higher temperature (Supplementary Table 2). And the three Micrarchaeota members from Fankou AMD sediment tended to have higher relative abundance in the lower layers, which were characterized by a higher Fe^2+^ concentration and lower Fe^2+^/Fe^3+^ ratio, while the only Parvarchaeota member from these layers (FK_Sedi_55_70) showed the opposite trend (Supplementary Fig. 5 and Supplementary Table 2).

### Comparative genomic analysis based on KEGG Orthology

**Fig. 2:**
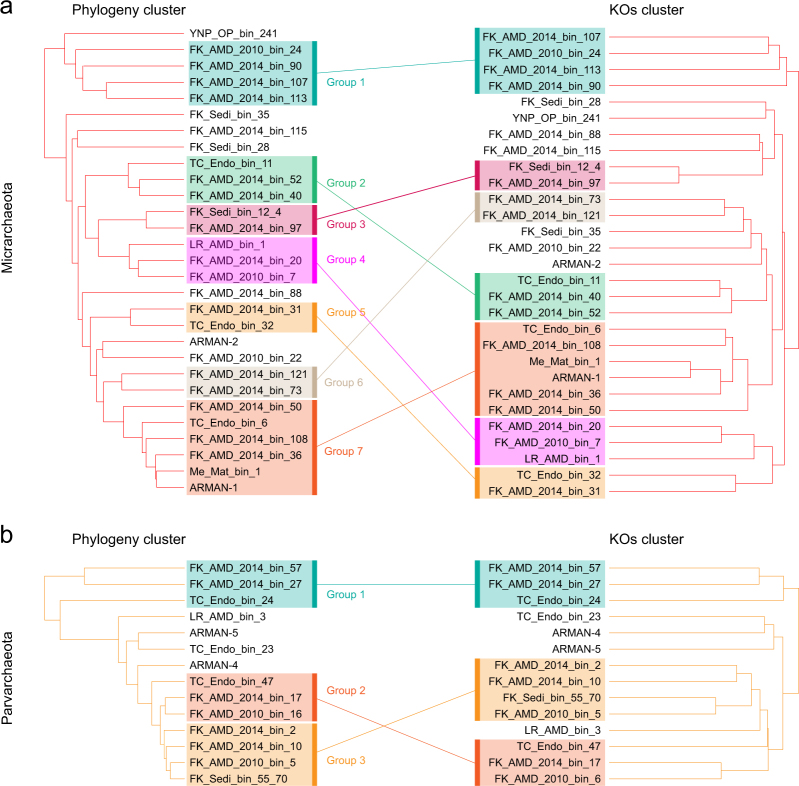
Comparative analyses of gene contents of Micrarchaeota and Parvarchaeota genomes. For **a** Micrarchaeota and **b** Parvarchaeota, the phylogeny cluster pattern (from Fig. 1) and the corresponding KEGG Orthology clustering pattern (based on occurrence of KOs in each genome; see Materials and methods) were compared, and the genomes were manually assigned to several clades/groups based on the cluster patterns. The same clade/group in a phylogeny cluster and gene contents clusters were linked with a solid line

To reveal if there are any clade specific metabolisms for both phyla, a hierarchical clustering analysis was performed based on the occurrence of KEGG Orthology (KO) patterns in each genome (Fig. 2) (Materials and methods). Compared to the phylogeny analyses results shown in Fig. 1 (left panels of Figs. 2a, b), different clades in both phyla generally have their unique gene contents, indicating variations in metabolic potentials between clades. For Micrarchaeota, the non-oxidative pentose phosphate pathway, aspartate-semialdehyde dehydrogenase, saccharopine dehydrogenase and zinc transporter genes were found only in group 1, Carbon monoxide dehydrogenase CooS and arginase genes occurred only in group 3, and ammonium transporter genes were only detected in group 7 (Fig. 2a). For Parvarchaeota, glutamine synthetase and L-asparaginase genes were exclusively found in group 1 and heptose III glucuronosyltransferase and inositol transporter genes in group 3 (Fig. 2b). It is reasonable to speculate that the specific gene contents of different clades may help them to inhabit distinct niches when they coexist with each other. A similar trend has been observed for members of Thermoplasmatales AMD archaea, the “alphabet plasmas”, which were also detected in our metagenomic samples. It has been found that the Thermoplasmatales AMD archaea differentiate by subtle genomic differences that allow their co-existence even if they share a great number of metabolic capabilities [49].

### Metabolic potentials of Micrarchaeota and Parvarchaeota

In order to resolve the physiological capabilities encoded in these genomes (including the four published ARMAN genomes), we used a variety of functional gene database comparisons for annotation (Materials and methods). Though the metabolisms of Micrarchaeota and Parvarchaeota have been previously predicted based on three genomes [11]; with a higher number of genomes representing higher phylogenetic diversity, we now have a better understanding of their metabolic versatility. The metabolic potentials of Micrarchaeota and Parvarchaeota based on all 43 genomes are detailed in the following several sections.

#### Cell membrane biosynthesis

Isoprenoids are essential in all living organisms, and vital for cell wall and membrane biosynthesis. As detected in other archaea, Micrarchaeota possess genes involved in the mevalonate pathway for biosynthesis of isoprenoid precursors (i.e., isopentenyl diphosphate and dimethylallyl diphosphate; IPP and DMAPP) [50]. Additionally, the presence of isopentenyl phosphate kinase genes indicates that they also have the alternative pathway for isopentenyl diphosphate synthesis (Supplementary Fig. 9). Notably, no genes of the mevalonate pathway or the methylerythritol 4-phosphate pathway [51] for IPP and DMAPP biosynthesis were detected in Parvarchaeota. However, considering their high genome completeness (Supplementary Table 4), it is possible that these pathways are present and the related genes too novel to be identified via sequence similarity comparison. Alternatively, they may obtain isoprenoids from the environment.

#### Stress reponse

**Fig. 3:**
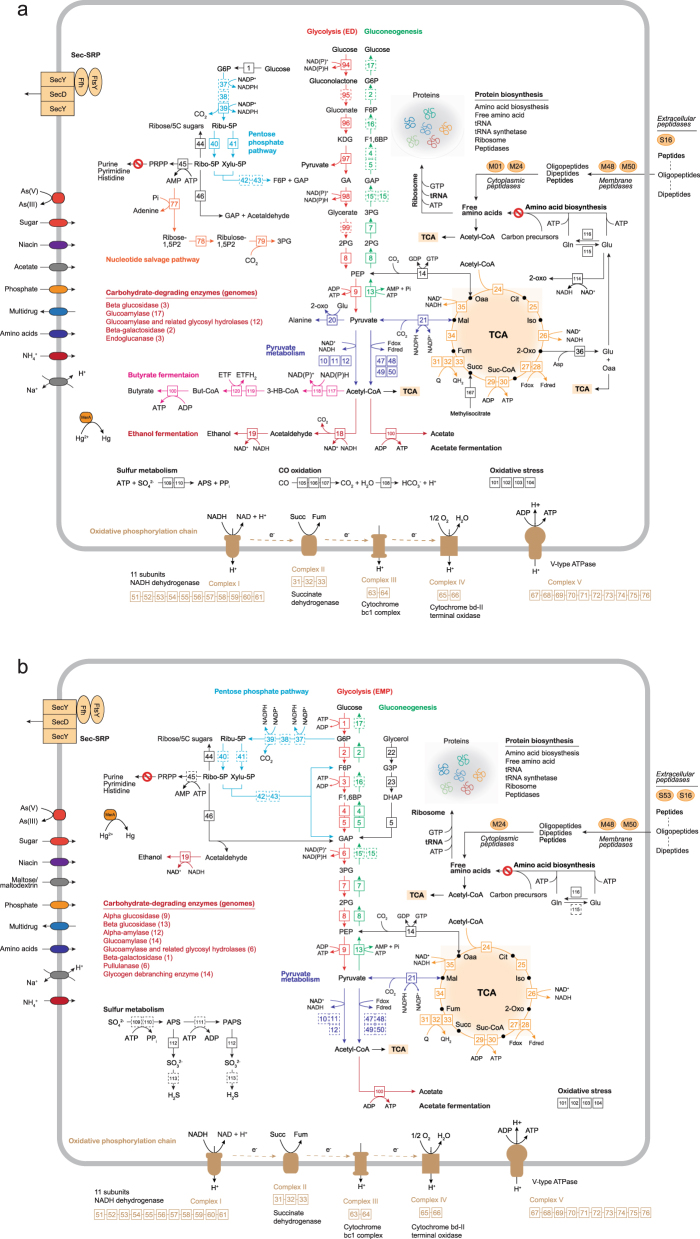
Overview of potential metabolic capabilities. Metabolic pathways were constructed based on the annotation of predicted genes (Materials and methods) and shown for **a** Micrarchaeota and **b** Parvarchaeota taxa. The glycolysis and gluconeogenesis pathways, the pentose phosphate pathway, the pyruvate metabolism, beta-oxidation of fatty acids, the TCA cycle and oxidative phosphorylation chain, protein biosynthesis-related pathways, membrane transporters, and other significant metabolisms are shown. The corresponding enzymes are represented by an ID in the figure and Supplementary Table 8 contains the gene copy number of each enzyme as well as of transporters, carbohydrate-degrading enzymes and peptidases. G6P glucose 6-phosphate, F6P fructose 6-phosphate, F1,6BP fructose 1,6-bisphosphate, GAP glyceraldehyde-3-phosphate, 3PG 3-phosphoglycerate, 2PG 2-phosphoglycerate, PEP phosphoenolpyruvate, KDG 2-keto-3-deoxygluconate, GA glyceraldehyde, G3P glycerol-3-phosphate, DHAP dihydroxyacetone phosphate, Ribu-5P ribulose 5-phosphate, Xylu-5P xylulose 5-phosphate, Ribo-5P ribose-5-phosphate, PRPP phosphoribosyl pyrophosphate, Oaa oxaloacetate, Cit citrate, Iso isocitrate, 2-Oxo 2-oxoglutarate, Suc-CoA succinyl-CoA, Succ succinate, Fum fumarate, Mal malate, Glu glutamate, Gln Glutamine, Fd ferredoxin, 3-HB-CoA 3-hydroxybutyryl-CoA, But-CoA Butyryl-CoA, As arsenic

Many genes related to environmental resistance were detected in both phyla, including those for drugs, antibiotics, heat shock, heavy metals, and oxidative stress (Fig. 3 and Supplementary Table 8). Interestingly, 15 Micrarchaeota and 10 Parvarchaeota genomes were detected with genes encoding resistance protein for fosmidomycin, which is an inhibitor of isoprenoid biosynthesis [52]. Almost all genomes of both phyla carry a superoxide dismutase and an alkyl hydroperoxide reductase allowing for a quick response to oxidative stress. Notably, Micrarchaeota carry a Fe and the Parvarchaeota carry a Mn superoxide dismutase, likely indicating their distinct evolutionary histories of obtaining this function. Interestingly, evidence of Micrarchaeota acting as a pathogen targeting other Bacteria was detected in three genomes that encode for a lytic murine transglycosylase [4], which could be transported outside of the cell via the Sec-SRP secretion system (Fig. 3a).

#### Amino acid and nucleotide biosynthesis

Although Micrarchaeota are able to generate alanine and glutamate respectively from pyruvate and aspartate (Fig. 3a), these organisms lack biosynthetic pathways for other amino acids, and Parvarchaeota lack biosynthetic pathways for all amino acids. However, both phyla encode for a variety of extracellular (e.g., S16, S53), membrane (e.g., M48, M50), and cytoplasmic (e.g., M01, M24) peptidases and transporters (e.g., amino acid permease) potentially allowing them to scavenge amino acids from the environment and thus to contribute to nitrogen cycling (Fig. 3 and Supplementary Table 8). Both phyla appear to lack genes for de novo biosynthesis of nucleotides from phosphoribosyl pyrophosphate (PRPP) (Supplementary Fig. 10), albeit some Micrarchaeota might produce PRPP from ribose-5-phosphate (Fig. 3a). However, complete pentose phosphate pathways (for ribose-5-phosphate generation) were not detected in these phyla, though some Micrarchaeota genomes harbored genes for the non-oxidative pentose phosphate pathway. To date, complete pentose phosphate pathways in Archaea have been detected in only two Nanohaloarchaea [53, 54] and two Woesearchaeota [3]. Consequently, both phyla may acquire nucleotides from the environment and/or other community members, for survival and cell proliferation, though free nucleotides may be very unstable in an acidic habitat where Micrarchaeota and Parvarchaeota are often found (pH value 0.5–4.0; Supplementary Table 2).

#### Carbon fixation

None of the six known carbon fixation pathways/cycles was detected in the Micrarchaeota and Parvarchaeota genomes, likely indicating that both phyla are heterotrophic. However, the ribulose 1,5-bisphosphate carboxylase/oxygenase (RubisCO), which is integral to the fixation of carbon dioxide, were detected in two Micrarchaeota genomes (Fig. 3a and Supplementary Table 8). The genes encoding for RubisCO, adenosine monophosphate (AMP) phosphorylase and ribose-1,5-bisphosphate isomerase were syntenic in these two genomes, indicating these RubisCOs likely function in a pathway for AMP metabolism as detected in other archaea [55].

#### Carbohydrate degradation

Genes encoding carbohydrate-degrading enzymes (glycoside hydrolases; GH) were detected in 41 out of 43 genomes (Fig. 3 and Supplementary Table 8), including the alpha-glucosidase (EC3.2.1.20; GH31) for degradation of starch and disaccharides, beta-glucosidase (EC3.2.1.21; GH1 and GH39) for disaccharides degradation, glucoamylase (EC3.2.1.3; GH15), alpha-amylase (EC3.2.1.1; GH57), and pullulanase (EC3.2.1.135; GH13) for starch degradation, endoglucanase (EC3.2.1.4) for cellulose degradation, and also a glycogen-debranching enzyme. Among them, GH1, GH15 and GH39 families were detected in both phyla, while GH13, GH31, and GH57 families were only detected in Parvarchaeota. This indicates that both phyla can degrade and utilize complex carbon sources from the environment, and that Parvarchaeota may be more versatile with more available related genes. Additionally, we detected that several Parvarchaeota contained genes for glycerol utilization such as the glycerol-3-phosphate dehydrogenase and glycerol kinase (Fig. 3b).

#### Glycolysis and gluconeogenesis

The glycolytic pathway was present, while gluconeogenesis was absent in both phyla (Fig. 3). A modified nonphosphorylative Entner–Doudoroff (ED) pathway has been described in other acidophilic archaea [56–58]. Several ED pathway related genes were found in 12 of the Micrarchaeota genomes, including the glucose dehydrogenase, gluconate dehydratase, 2-dehydro-3-deoxy-D-gluconate aldolase, and glyceraldehyde dehydrogenase (Fig. 3a). Though the pathway was incomplete, these genes were syntenic, indicating that the missing genes were undetectable due to novelty or these archaea are using an alternative pathway. A unique Embden–Meyerhof–Parnas (EMP) glycolysis pathway was detected in all Parvarchaeota (Fig. 3b), which utilize NAD(P)-dependent nonphosphorylating glyceraldehyde-3-phosphate dehydrogenase for the oxidation of glyceraldehyde-3-phosphate to 3-phosphoglyceric acid, without the formation of 1,3-bisphosphoglyceric acid and ATP [59]. Similar activities have been detected in other thermoacidophiles [60, 61]. For both phyla, their glycolysis pathways could not generate ATP with only NAD(P)H for anabolism, indicating the significance of alternative ATP generation ways for their activity and growth.

#### Pyruvate metabolism

Micrarchaeota are able to utilize pyruvate by converting pyruvate to acetyl-CoA using the pyruvate dehydrogenase complex (in 25 genomes) and pyruvate:ferredoxin oxidoreductase (in one genome). It is surprising that these genes were absent in Parvarchaeota, considering they have the potential to use multiple carbon resources (e.g., starch, disaccharides; see above) to generate pyruvate (Fig. 3b). However, the 2-oxoglutarate/2-oxoacid ferredoxin oxidoreductase (OFOR) detected in Parvarchaeota may convert pyruvate to acetyl-CoA, as evidenced in *Sulfolobus tokodaii* strain 7 [62]. Otherwise, Parvarchaeota may transfer pyruvate into the TCA cycle via phosphoenolpyruvate synthase and phosphoenolpyruvate carboxykinase (Fig. 3b). Moreover, Parvarchaeota genomes encoded for a cytoplasmic ferredoxin (Supplementary Table 8), which may have the potential to convert pyruvate.

#### TCA cycle

Distinct from other DPANN members [3], a complete or near-complete TCA cycle was identified in both phyla (Fig. 3). All but one Micrarchaeota genome lacked genes encoding succinyl-CoA synthetase transferring succinyl-CoA to succinate in the TCA cycle, however, the detected malate dehydrogenase (oxaloacetate decarboxylating) may generate malate from pyruvate. Additionally, a methylisocitrate lyase gene was detected in seven Micrarchaeota genomes (Fig. 3a), and may generate succinate from methylisocitrate to complete the TCA cycle. Interestingly, both phyla have a two-subunit type OFOR for converting 2-oxoglutarate to succinyl-CoA, which is often utilized by microaerophilic and strictly anaerobic organisms for redox coupling of ferredoxin [63].

#### Aerobic respiration

A NADH dehydrogenase was detected in both phyla, however, lacking the NADH-binding module (encoded by nuoEFG) (Fig. 3), a feature has been observed in some bacteria [64, 65] and all Thermoplasmatales [45]. Such a NADH dehydrogenase was suggested to accept electrons from reduced ferredoxin produced by pyruvate ferredoxin oxidoreductase or 2-oxoglutarate ferredoxin oxidoreductase enzymes to generate a proton motive force [65]. Accordingly, the succinate dehydrogenase/fumarate reductase (complex II) may be used as electron inflow into the respiratory chain, as proposed in *Cuniculiplasma divulgatum* [45]. A cytochrome bc1 complex was detected as complex III, while the absence of the iron-sulfur subunit in most genomes (only detected in two Micrarchaeota and one Parvarchaeota), raise doubts in its involvement in a respiratory chain. Both phyla contained a cytochrome bd-II terminal oxidase, which was reported to be induced under O_2_ limiting conditions as cytochrome bd-I [66], and other terminal oxidase genes were absent. A V-type H^+^-transporting ATP synthase containing 9 subunits was encoded in both phyla for ATP production.

#### Fermentation

Based on their gene content both phyla have the capacity to ferment and respire aerobically (Fig. 3 and Supplementary Table 8). Most Micrarchaeota likely are able to ferment alcohol via the aldehyde dehydrogenase and alcohol dehydrogenase (Fig. 3a). Conversely, only alcohol dehydrogenase genes were detected in Parvarchaeota, which may use the acetaldehyde produced via deoxyribose-phosphate aldolase as substrate (Fig. 3b). Additionally, evidence for butyrate fermentation were also detected in 16 Micrarchaeota genomes (Fig. 3a). Genes encoding an ADP-forming acetyl-CoA synthetase, which is responsible for acetate production and ATP generation from acetyl-CoA [67], were detected in both phyla, indicating their potential in acetate fermentation (Fig. 3).

#### Potential iron oxidation

**Fig. 4:**
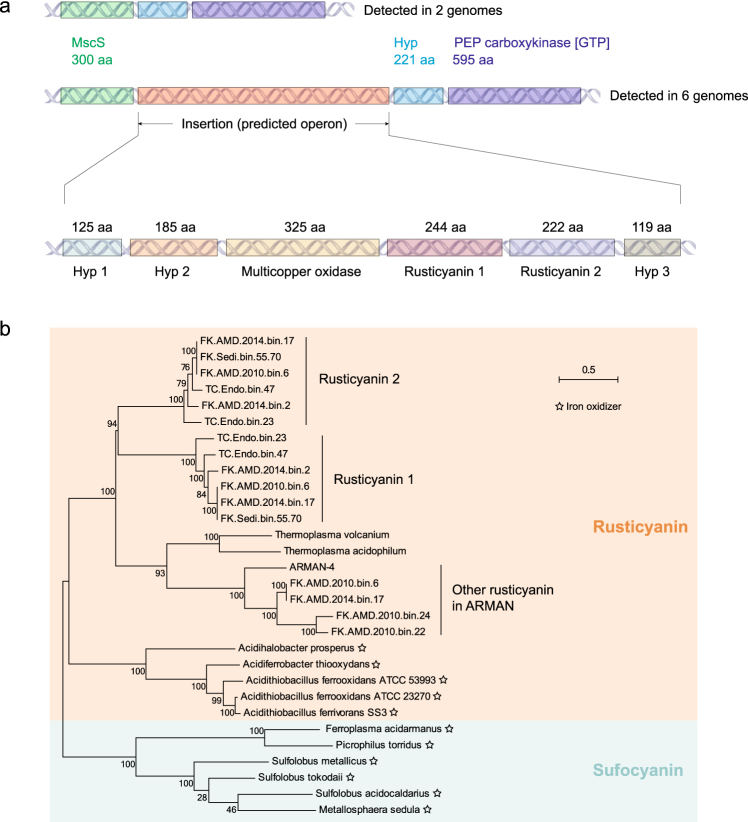
Cluster of rusticyanin related genes detected in Parvarchaeota. **a** A rusticyanin cluster was detected in 6 Parvarchaeota genomes between a MscS (small-conductance mechanosensitive channel-like) gene and a hypothetical protein and a PEP (phosphoenolpyruvate) carboxykinase gene (middle panel). No inserted cluster was found in other Parvarchaeota (above panel). The cluster includes two rusticyanin proteins, one multicopper protein and three hypothetical proteins (bottom panel). The amino acid length of all proteins are shown. **b** Phylogeny analyses of rusticyanin protein sequences encoded in ARMAN (those two in the cluster and others detected in ARMAN) and genomes of confirmed iron oxidizers, also including the similar key iron oxidase of sulfocyanin. The tree was built using MEGAN (version 7.0.14) using Maximum Likelihood method with 100 replicates, bootstrap numbers are shown

Interestingly, we detected an operon containing homologous proteins of rusticyanin (copper blue) and multicopper oxidases in six Parvarchaeota genomes (Fig. 4a). Structural prediction indicated two hypothetical proteins in the operon, a multicopper oxidase and one rusticyanin containing a transmembrane motif, while the other rusticyanin was a periplasmic or extracellular protein, and the third hypothetical protein was located in the cytoplasm (Supplementary Table 9). Similar rusticyanin proteins have been shown to be involved in iron oxidation in *Acidithiobacillus ferrooxidans* [68] (Fig. 4b). Considering the high availability of ferrous iron in the AMD environments populated by Parvarchaeota (Supplementary Table 2), they are likely involved in iron oxidation, which is speculative and requires experimental confirmation.

As stated above, genomic analyses of Micrarchaeota and Parvarchaeota revealed that several central metabolic pathways were absent. Both phyla lacked biosynthetic pathways of amino acids with the exception of Micrarchaeota that seems to be able to synthesize alanine and aspartate. Additionally, both phyla did not encode genes for de novo biosynthesis of nucleotides, and only few pentose phosphate pathway genes were detected. Gluconeogenesis was absent in both phyla, several nonphosphorylative ED glycolysis pathway related genes were detected in 12 Micrarchaeota genomes, however, it is so far unclear whether the pathway was complete. In comparison, a full EMP glycolysis pathway was detected in all Parvarchaeota genomes. The TCA cycle was detected to be near complete in all Micrarchaeota genomes (except in FK_AMD_2014_bin_31 and TC_Endo_bin_32). Notably, only FK_AMD_2014_bin_90 contained the gene encoding succinyl-CoA synthetase, while this gene and a complete or near-complete TCA pathway was detected in all Parvarchaeota genomes. Relatedly, parts of the aerobic respiration chain including a cytochrome bc1 complex and a cytochrome bd-II terminal oxidase were detected in both phyla, while the NADH dehydrogenase and succinate dehydrogenase genes were absent in the two abovementioned Micrarchaeota genomes without the TCA cycle. Additionally, no isoprenoid precursors biosynthesis pathway genes were detected in Parvarchaeota, thus hindering the membrane biosynthesis. In summary, the disabilities of both phyla to synthesize amino acids and nucleotides are likely their largest obstacle for their growth, and these molecules may thus be obtained from those in environments and/or other community members.

### Laboratory enrichment of Micrarchaeota and Parvarchaeota

**Fig. 5:**
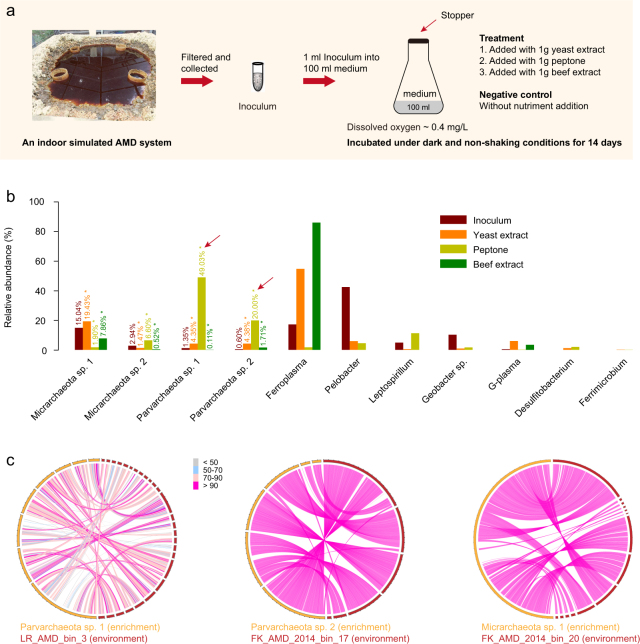
Laboratory enrichment of ARMAN from a simulated AMD system. **a** Diagram showing the experimental design of the enrichment experiment (Materials and methods). The dissolved oxygen concentration was determined when collecting enriched cells. **b** Relative abundance of taxa in the inoculum and enrichments with different nutriments. The exact numbers for ARMAN taxa are shown, and those from MDA metagenomes are indicated by asterisks. **c** Circos-based alignment of genomes from enriched communities against those from environmental samples reported in this study. The alignment of Micrarchaeota sp. 2 contained too many scaffolds and is not shown. Each scale on the scaffold represents a length of 20 kbp

When Micrarchaeota and Parvarchaeota genomes were first reported [11], they were thought to be aerobic based on their high levels of succinate dehydrogenase in the proteomic pool. However, Ziegler et al. found that ARMAN spp. were detected only to inhabit the anoxic niches of an acidic snottite, indicating they may be anaerobic, which was documented by anaerobic enrichment [17]. In this study, our broader genomic sampling of both phyla suggests a microaerobic or anaerobic lifestyle based on two clues, (a) an aerobic respiratory chain and fermentation potential were detected, (b) genomes of both phyla encode OFOR and the terminal oxidase cytochrome bd-II, which are often utilized by microaerophilic and strictly anaerobic organisms. To confirm this speculation, we performed enrichments under microaerobic/anaerobic conditions (dissolved oxygen, ~0.4 mg/L) provided with different nutriments (Fig. 5a; see Materials and methods for details).

ARMAN were detected in all the three enrichments supplemented yeast extract, peptone, and beef extract, respectively, while found absent in the control treatment lacking nutriment addition (Supplementary Fig. 4). This suggests that ARMAN spp. either could not grow in medium without additional nutriment, or were below the detection level of 16S rRNA gene PCR analyses. These results suggested that ARMAN can grow in the three enrichments added with nutriments. Due to the low biomass of enriched communities (likely due to the relatively short growth period of 14 days), we used MDA prior to shotgun metagenome sequencing. Metagenomic co-assembly and genome binning of the three enriched communities obtained two Micrarchaeota (Micrarchaeota sp. 1 and sp. 2) and two Parvarchaeota genomes (Parvarchaeota sp. 1 and sp. 2) (Fig. 5b and Supplementary Fig. 11). These enriched genomes showed a high sequence similarity to the genomic bins from the environments, with average amino acid identity (AAI) from 95.2–97.0%. One exception was the Parvarchaeota sp. 1 which exhibited a lower AAI of 77.4% with LR_AMD_bin_3 (Fig. 5c).

As it was reported that MDA may skew the relative abundance analyses [46], we assessed this bias by comparing the non-MDA inoculum community with its MDA data (in triplicates) (Materials and methods). The three MDA replicates yielded highly consistent results (low SE values; Supplementary Table 10) while many of the taxa with relative abundance <0.5% in the non-MDA sample could no longer be detected in the MDA samples. The two Micrarchaeota spp. and the two Parvarchaeota spp. all tended to have higher relative abundance values in the amplified samples (Supplementary Table 10). In details, Micrarchaeota sp. 1 raised from 15.04% in the non-MDA sample to 17.85% on average in the three MDA replicates, Micrarchaeota sp. 2 from 2.94 to 4.34% on average, Parvarchaeota sp. 1 from 1.35 to 2.76% on average, and Parvarchaeota sp. 2 from 0.60 to 0.72% on average.

For those species in the enrichments, Micrarchaeota sp. 1 has a slightly higher abundance only in the yeast extract enrichment, which may be a result of the MDA treatment as stated above. However, Parvarchaeota sp. 1 and sp. 2, both have a much higher relative abundance in the peptone enrichment (49.03 and 20.00%, respectively), as compared to the inoculum (1.35 and 0.60%, respectively) (Fig. 5b). Such dominance by the two Parvarchaeota spp. does not seem to be a result of the MDA step. Thus, the water-soluble mixture of polypeptides and amino acids in peptone may be favored by these Parvarchaeota members for their growth in laboratory condition. Additionally, two of the enriched communities were detected with the strict anaerobic microorganism of *Desulfitobacterium* (Fig. 5c), which was neither observed in the non-MDA community nor in MDA inoculums, indicating the enrichments also accelerated the growth of other anaerobes.

Altogether, all these results abovementioned supported the growth of ARMAN spp. in these enrichments under microaerobic/anaerobic condition. Due to the low biomass of the enriched communities, more direct approaches such as fluorescence in situ hybridization and imaging-based cell quantifications could not be performed. Extended enrichment time period and/or successive transfers are needed in future studies for a better understanding of their physiology and metabolisms.

### Current knowledge of connection between Micrarchaeota spp. and Thermoplasmatales members

**Fig. 6:**
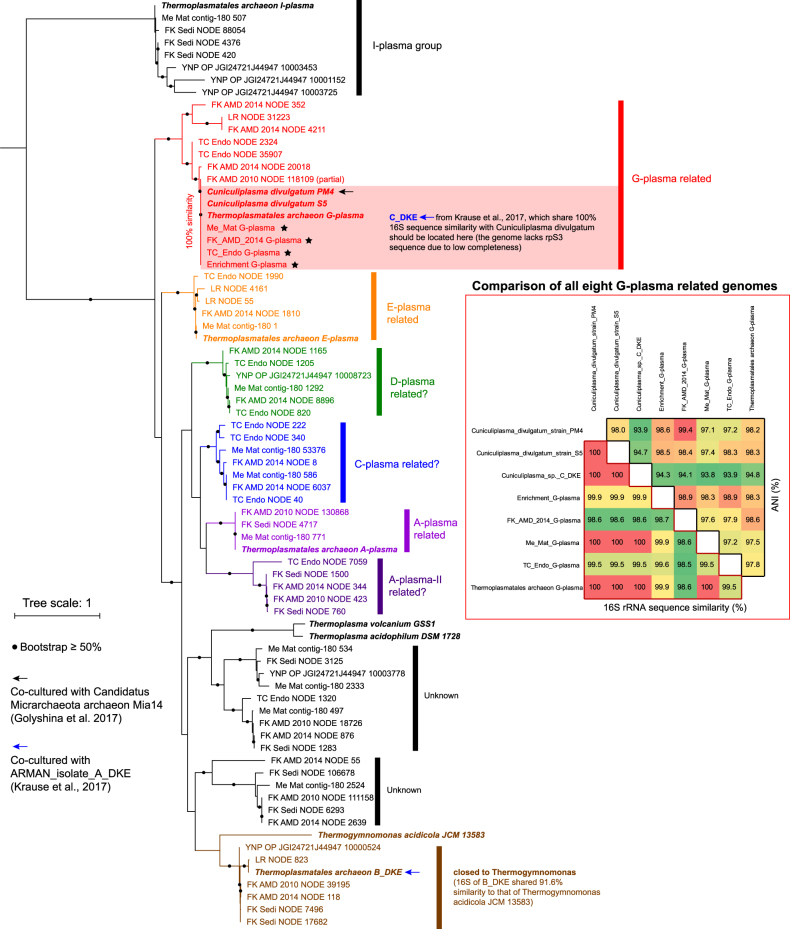
Phylogenetic analyses of Thermoplasmatales related taxa detected in the microbial communities analyzed in this study. The tree was built using all available Thermoplasmatales related rpS3 sequences detected in the metagenomes, and those of related published genomes (in italic bold) and included A-plasma, I-plasma, E-plasma, G-plasma (also *Cuniculiplasma* spp.; four genomes in total, while C_DKE lacks rpS3 due to low completeness), *Thermoplasma volcanium*, *Thermoplasma acidophilum*, *Thermogymnomonas acidicola*, and Thermoplasmatales archaeon B_DKE. For a comparison, the G-plasma related spp. in the analyzed communities sharing 100% rpS3 sequence similarity with published genomes, were binned to obtain their genomes (indicated by stars), and these eight G-plasma related genomes share high 16S rRNA simiarity (98.5–100%) and ANI (94.1–99.4%)

During the review course of this manuscript, two stable co-cultures of Micrarchaeota sp. and Thermoplasmatales were reported from samples from AMD-related environments [69, 70]. Krause et al. obtained a consortium after 2.5 years of successive transfers in an anoxic medium [70]. This consortium included one Micrarchaeota sp. (A_DKE), one *Cuniculiplasma divulgatum* related Thermoplasmatales (C_DKE; sharing 100% 16S rRNA gene sequence similarity with *Cuniculiplasma divulgatum*), another Thermoplasmatales member related to *Thermogymnomonas acidicola* (sharing 91.6% 16S sequence similarity) and a fungus (Fig. 6 and Supplementary Fig. 12). CARD-FISH showed A_DKE cells were mostly located in cell agglomerates formed by B_DKE and/or C_DKE. The host of A_DKE could not be determined because no stable co-culture could be obtained that included only A_DKE and one of the two Thermoplasmatales. More recently, Golyshina et al. reported the dependent host of a Micrarchaeota sp. (Mia14) was an isolated Thermoplasmatales sp., *Cuniculiplasma divulgatum* PM4 (Fig. 6 and Supplementary Fig. 12), by using CARD-FISH analyses and thereby confirmed their interaction [69].

Notably, Micrarchaeota Mia14 lacks the TCA cycle and the respiratory chain is incomplete and consisted only of a V-type H^+^-transporting ATP synthase and cytochrome bd-II terminal oxidase, thus it remains unclear if Micrarchaeota Mia14 can generate energy by itself, even though it contains an acetyl-CoA synthetase that could generate energy over substrate level phosphorylation. This is consistent with our findings for FK_AMD_2014_bin_31 and TC_Endo_bin_32, which clustered with Micrarchaeota Mia14 on phylogeny (Supplementary Fig. 12a). Additionally, Mia14 shares a 16S rRNA gene sequence similarity of 95.5% with TC_Endo_bin_32 (no 16S rRNA gene was binned for FK_AMD_2014_bin_31), indicating they belong to the same genus [36] (Supplementary Fig. 12b). Altogether, these results suggest that the absence of these pathways could be linked to a specific phylogenetic clade of Micrarchaeota.

To date, three experimentally validated examples of interactions between small-sized archaea and their hosts have been reported, *Nanoarchaeum equitans* and *Ignicoccus hospitalis* [71], *Nanoarchaeota* Nst1 and *Sulfolobales* spp. [72], and *Candidatus* Nanopusillus acidilobi and *Acidilobus* spp. [5]. All these small archaea lack most primary biosynthesis pathways, and more importantly, depend on their hosts for energy production, which is likely a key point for such close relationships between these species (“ectoparasitic lifestyle”). Thus, it is important to understand why Micrarchaeota Mia14 (and related group members) lack the whole TCA cycle and contain only some components of respiratory chain, and two distinct scenarios of evolution are proposed. The first one is that, the lowest common ancestor (LCA) of all Micrarchaeota clades analyzed in this study harbored the TCA cycle and complete respiratory chain, which were retained by most of the genomes during their evolution history, except for Micrarchaeota Mia14. And the second, the LCA lacked the TCA cycle and respiratory chain, and most Micrarchaeota clades gained these functionalities during their evolution history, while the Micrarchaeota Mia14 related clade only obtained part of them.

To distinguish between these two scenarios, we searched for homologs involved in energy generation by searching for related proteins in NCBI and subsequently performed phylogenetic analyses (Materials and methods). First, we analyzed energy generation related components shared by Micrarchaeota Mia14 and other Micrarchaeota spp., that is, V-type H^+^-transporting ATP synthase and cytochrome bd-II terminal oxidase and acetyl-CoA synthetase. To reconstruct their evolution history, homologs searching of related proteins in NCBI and subsequent phylogenetic analyses were performed (Material and methods). Phylogenetic analyses of ATP synthase subunit D showed all Parvarchaeota and most Micrarchaeota spp. clustered with Thermoplasmatales members. Interestingly, seven Micrarchaeota spp. (five of them were from Family 1 Micrarchaeota; Supplementary Fig. 7) showed a distinct evolutionary history with different genetic structure of the ATP synthase complex (Supplementary Fig. 13a), and all these members lacked the cytochrome bd-II terminal oxidase. For Mia14 and 16 Micrarchaeota and 13 Parvarchaeota genomes that encoded a cytochrome bd-II terminal oxidase, a phylogenetic analyses of both subunits (I and II) consistently revealed their evolutional connections with those from Thermoplasmatales (Supplementary Fig. 13b, c). For the acetyl-CoA synthetase, all 33 Micrarchaeota genomes contained both the alpha chain and beta chain, while both subunits were fused as a single gene in Parvarchaeota, which has been reported in Thermoplasmatales such as G-plasma [45]. A phylogeny analysis of the acetyl-CoA synthetase showed that Micrarchaeota clustered most closely to these proteins in other DPANN members (Supplementary Fig. 13d), and the phylogenetic pattern was highly consistent with the taxonomic phylogeny shown in Fig. 1, indicating this functionality was gained by the LCA of all these Micrarchaeota clades. While Parvarchaeota clustered with Methanocaldococcus spp. and only distantly related to Thermoplasmatales (Supplementary Fig. 13d).

Next, we investigated the phylogeny of other TCA cycle and respiratory chain proteins absent in Micrarchaeota Mia14, such as NADH dehydrogenase, succinate dehydrogenase, citrate synthase, aconitate hydratase and others. The succinate dehydrogenase was analyzed based on its flavoprotein subunit and this revealed that, Micrarchaeota spp., ARMAN-2 and all Family 1 Micrarchaeota members (Supplementary Fig. 7) and was most closely related to sequences from *Caldisphaera lagunensis* (Supplementary Fig. 13e), a thermoacidophilic Crenarchaeota species isolated from a hot spring in the Philippines (Itoh et al., 2003). The other Micrarchaeota and all Parvarchaeota spp. genomes clustered with Thermoplasmatales, indicating that Micrarchaeota spp. have experienced at least two different evolutionary events for the succinate dehydrogenase. Similarly, phylogenetic analyses of the citrate synthase and aconitate hydratase showed their close evolutional history with Thermoplasmatales (Supplementary Fig. 13e–g).

Overall, these phylogenetic analyses revealed that the evolutionary history of energy related pathways in ARMAN are very complex, especially for Micrarchaeota (Supplementary Figs. 13a–g). The current situation could be well explained by the second evolutionary scenario mentioned above, but this complex scenario might also be caused by several gain and loss events. These results provide first insights into the close evolutionary relationship between both Micrarchaeota and Parvarchaeota with Thermoplasmatales, which is consistent with their co-occurrence in all the analyzed communities (Fig. 6) and the two co-culture studies [69, 70]. Moreover, the G-plasma related species are likely the potential hosts of some ARMAN members [69, 70], and it is surprising to find their extremely high genomic sequence similarities when considering the large geographic distances (eight genomes shown in Fig. 6 were from Europe, Asia, North America, and South America). Our data suggest that most of the Micrarchaeota and Parvarchaeota members analyzed in this study “only” lacked the ability to synthesize amino acids and nucleotides, thus it is reasonable to speculate that they may depend on multiple potential hosts [69]. The availability of valuable co-cultures will provide ample opportunities to experimentally test these intriguing relationships in future studies.

## Concluding remarks

Ultra-small members of the DPANN superphylum are widely distributed [3–7], nevertheless cultured representatives are limited and their ecological roles are largely unknown. In this study, we have increased the genomic sampling of two DPANN phyla, Micrarchaeota and Parvarchaeota and thereby revealed their distribution in environments, and inferred their metabolic potentials. Although these Micrarchaeota and Parvarchaeota genomes are among the smallest archaeal genomes, they contained pathways for carbon, nitrogen, and iron cycling. The absence of biosynthesis pathways for amino acids and nucleotides indicated their potential dependence on co-occurring community members when nutrients are limited. Moreover, we were able to successfully enrich both phyla in high relative abundance under low O_2_ conditions in the laboratory, and comparative genomic analyses indicated the evolutionary history of several energy related proteins between both Micrarchaeota and Parvarchaeota phyla and Thermoplasmatales, providing first insights into their close relationship in nature and laboratory enrichments. Overall, this study reveals shared and specific features of novel genomes from little studied organisms related to the Micrarchaeota and Parvarchaeota lineages and provides new insights into the interactions and functioning of AMD and hot spring communities.

## Electronic supplementary material

Supplementary information

Supplementary Tables
