# Supplementary material for: Metabolic versatility of small archaea Micrarchaeota and Parvarchaeota
Source: ISME J. 2017 Dec 8;12(3):756–75. doi: 10.1038/s41396-017-0002-z (PMC5864196; doi:10.1038/s41396-017-0002-z)
Supplement: Supplementary file 1 — Supplementary information [file 41396_2017_2_MOESM1_ESM.pdf]

Supplementary information

for

Metabolic versatility of small archaea Micrarchaeota and Parvarchaeota

Lin-Xing Chen[1], Celia Méndez-García[2,3], Nina Dombrowski[4], Luis E. Servín-Garcidueñas[5], Emiley A. Eloe-Fadrosh[6], Bao-Zhu Fang[1], Zhen-Hao Luo[1], Sha Tan[1], Xiao-Yang Zhi[7], Zheng-Shuang Hua[1], Esperanza Martinez-Romero[8], Tanja Woyke[6], Li-Nan Huang[1], Jesús Sánchez[2], Ana Isabel Peláez[2], Manuel Ferrer[9], Brett J. Baker[4,*], Wen-Sheng Shu[10,*]

[1] State Key Laboratory of Biocontrol, Guangdong Key Laboratory of Plant Resources, College of Ecology and Evolution, Sun Yat-Sen University, Guangzhou 510275, PR China.

[2] Departamento de Biología Funcional-IUBA, Universidad de Oviedo, Oviedo, Spain.

[3] Carl R. Woese Institute for Genomic Biology, University of Illinois at Urbana-Champaign, Urbana, USA.

[4] Department of Marine Science, University of Texas Austin, Marine Science Institute, Port Aransas, Texas 78373, USA.

[5] Laboratory of Microbiomics, National School of Higher Studies Morelia, National University of Mexico, Morelia, Michoacan, 58190, Mexico.

[6] Joint Genome Institute, Walnut Creek, California 94598, USA.

[7] Yunnan Institute of Microbiology, Yunnan University, Kunming, 650091, People's Republic of China.

[8] Department of Ecological Genomics, Center for Genomic Sciences, National University of Mexico, Cuernavaca, Morelos, 62210, Mexico.

[9] Institute of Catalysis, Consejo Superior de Investigaciones Científicas (CSIC), Madrid, Spain.

[10] School of Life Sciences, South China Normal University, Guangzhou 510631, PR China

[*] Correspondence authors: W.S.S., shuws@mail.sysu.edu.cn; B.J.B., acidophile@gmail.com.

Running title: Metabolic versatility of ARMAN

Supplementary information includes supplementary tables, supplementary figures, and supplementary references. The tables are shown in another excel file, and the title of tables are listed in the next page. All the supplementray figures and their legends are shown in this file.

Supplementary Tables

All the Supplementary Tables were contained in a separate Excel file ("Supplementary Tables"), see below for Table titles, and see coresponding legends in the Excel file.

Supplementary Table 1 Genomic summary of available draft or complete genomes from the novel superphylum DPANN.

Supplementary Table 2 The geochemical characteristics of the sampling locations.

Supplementary Table 3 The information of metagenomic datasets and assembly results.

Supplementary Table 4 The occurences of 54 archaeal conserved singel copy genes (by Castelle et al., 2015) in genomes of Micrarchaeota and Parvarchaeota.

Supplementary Table 5 Informtaion of Candidatus Micrarchaeota and Parvarchaeota genome bins reconstructing from metagenomic datasets.

Supplementary Table 6 The number of KEGG Orthology detected in each ARMAN genome via KAAS

Supplementary Table 7 The information of retrieved Candidatus Micrarchaeota and Parvarchaeota associated 16S rRNA gene sequences

Supplementary Table 8 The number of genes assigned to metabolic pathways as shown in Figure 3 of the main text and others.

Supplementary Table 9 The information of genes in the poteintal iron oxidation gene operons detected in Parvarchaeota.

Supplementary Table 10 The relative abundance values for taxa obtained from non-MDA and MDA inoculum communities.

Supplementary references

Castelle, C. J. et al. Genomic expansion of domain archaea highlights roles for organisms from new phyla in anaerobic carbon cycling. Curr. Biol. 25, 690–701 (2015).

Dick, G. J. et al. Community-wide analysis of microbial genome sequence signatures. Genome Biol. 10, R85 (2009).

Ghai, R. et al. New abundant microbial groups in aquatic hypersaline environments. Sci. Rep. 1, 135 (2011).

Martínez-García M., Santos F., Moreno-Paz M., Parro V., Antón J. Unveiling viral-host interactions within the 'microbial dark matter'. Nat. Commun. 5, 4542 (2014).

Narasingarao, P. et al. De novo metagenomic assembly reveals abundant novel major lineage of Archaea in hypersaline microbial communities. ISME J. 6, 81–93 (2012).

Podar, M. et al. Insights into archaeal evolution and symbiosis from the genomes of a nanoarchaeon and its inferred crenarchaeal host from Obsidian Pool, Yellowstone National Park. Biol. Direct 8, 9 (2013).

Rinke, C. et al. Insights into the phylogeny and coding potential of microbial dark matter. Nature 499, 431-437 (2013).

Tyson, G. W. et al. Community structure and metabolism through reconstruction of microbial genomes from the environment. Nature 428, 37–43 (2004).

Waters, E. et al. The genome of Nanoarchaeum equitans: insights into early archaeal evolution and derived parasitism. Proc. Natl. Acad. Sci. USA 100, 12984–12988 (2003).

Wurch, L. et al. Genomics-informed isolation and characterization of a symbiotic Nanoarchaeota system from a terrestrial geothermal environment. Nature Commun. 7, (2016).

Supplementary Figures

Below are all **Supplementary Figures** and corresponding legends.

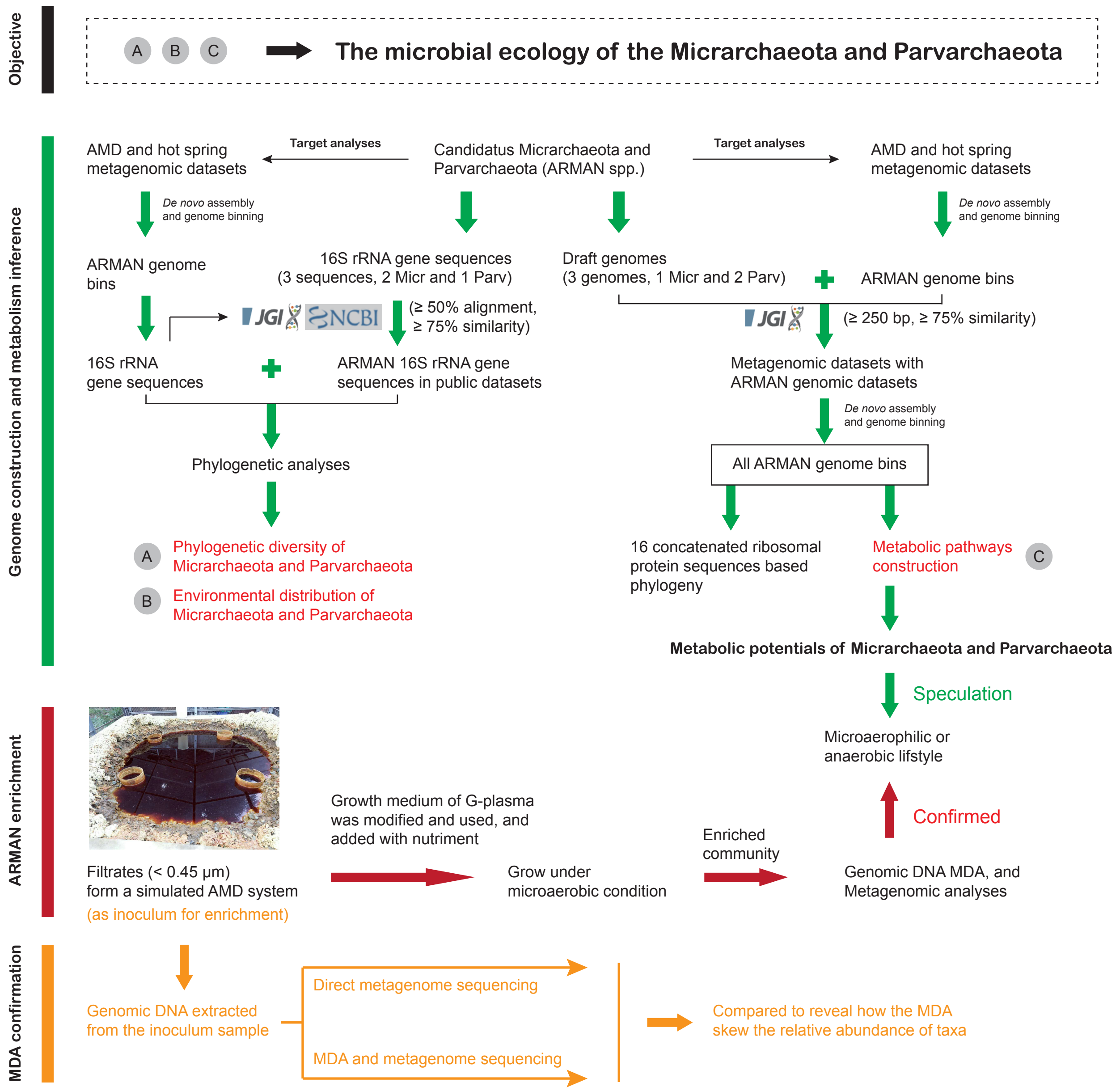

Supplementary Figure 1. The analyses pipeline of this study, including four separated parts, (1) The objective of this study, (2) the approaches to reconstruct genome bins and to infer metabolisms, (3) the enrichment experiment and (4) the influence of MDA on relative abundance analyses. Please see Materials and methods in the main text for details of each processing step.

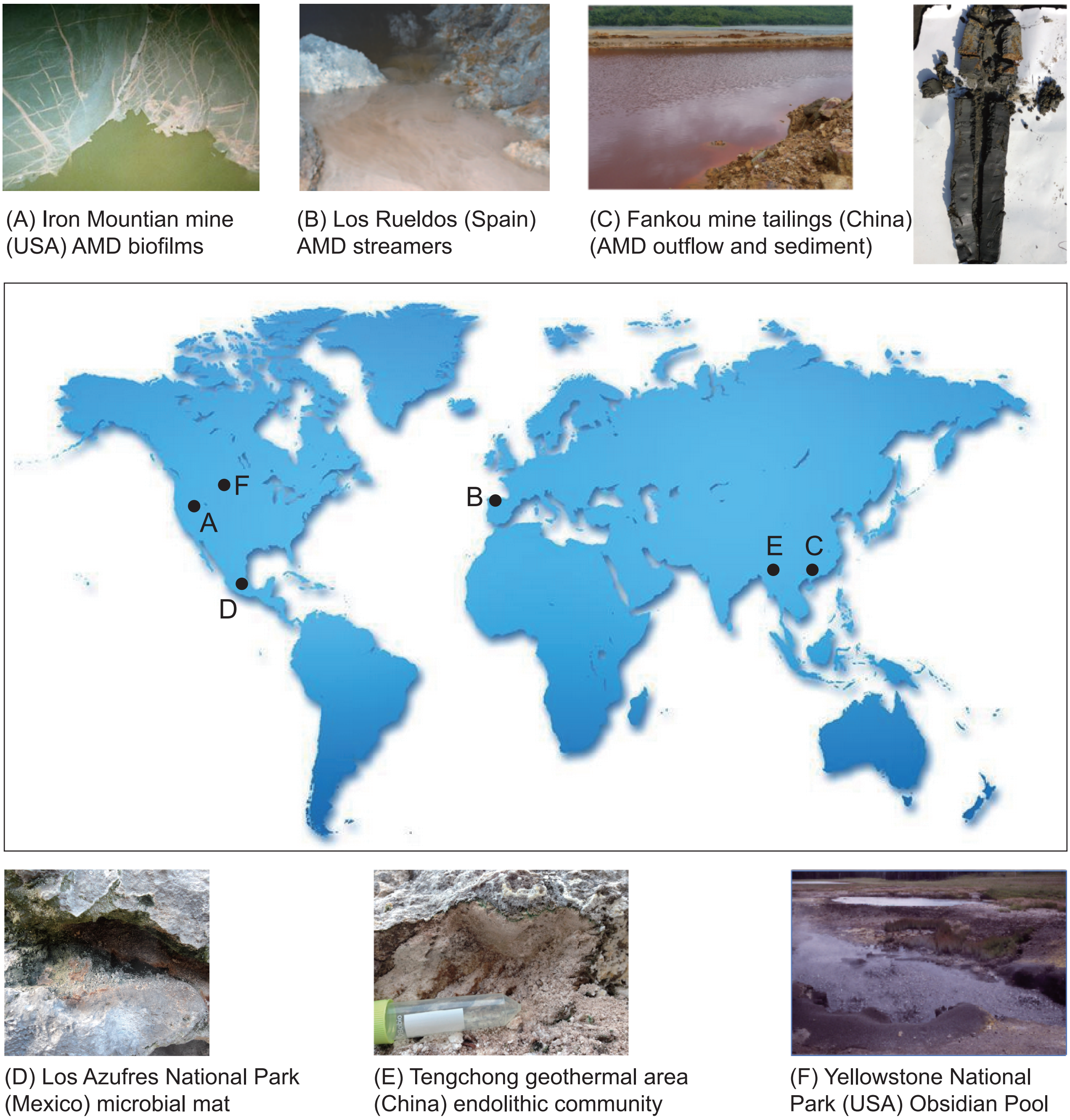

Supplementary Figure 2. The map shows the locations of the Iron mountain (A) where ARMAN were first described, and the two AMD (B,C) and three hot spring (D,E,F) from where ARMAN associated metagenome datasets analyzed in this study were obtained. (A) The AMD biofilms in Iron mountain of Richmond, California (Tyson et al., 2004). (B) the AMD streamers in Los Rueldos. (C) The AMD outflow and the AMD sediment core collected in the AMD pond on Fankou mine tailings. (D) The microbial mat in Los Azufres National Park. (E) The endolithic community sample collected in the "Drty" location of the Tengchong geothermal area. The 50 ml tube is for scale. (F) The Yellowstone National Park Obsidian Pool hot spring (this is an open metagenome at JGI/IMG database).

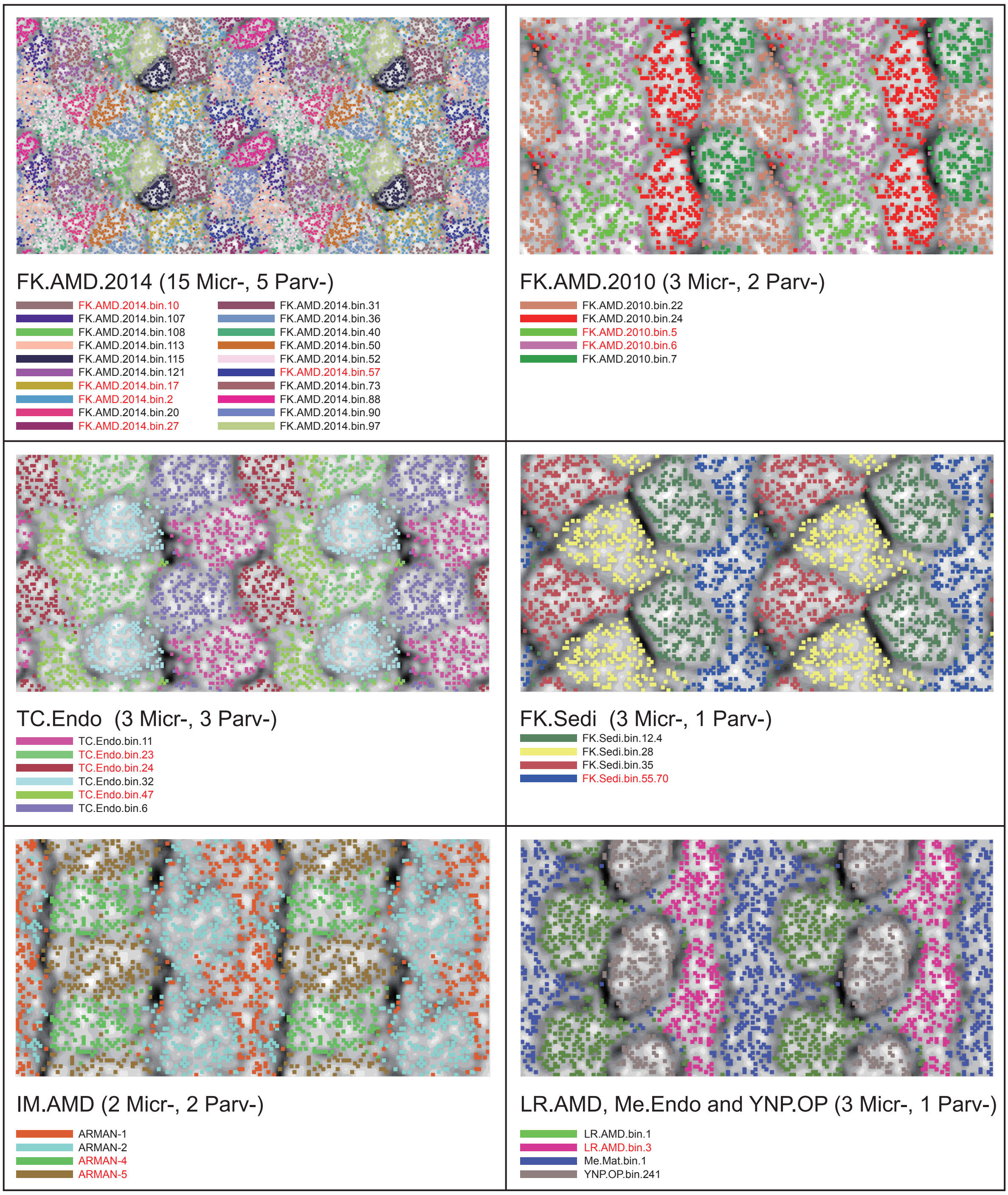

Supplementary Figure 3. ESOM (Emergent Self-Organizing Map) of ARMAN associated genome bins described in this study (including 29 Micrarchaeota genomes and 14 Parvarchaeota genomes). The ESOM analysis was performed as previously described (window = 5000 bp) (Dick et al., 2009). The bin names in black represent Micrarchaeota, and in red represent Parvarchaeota.

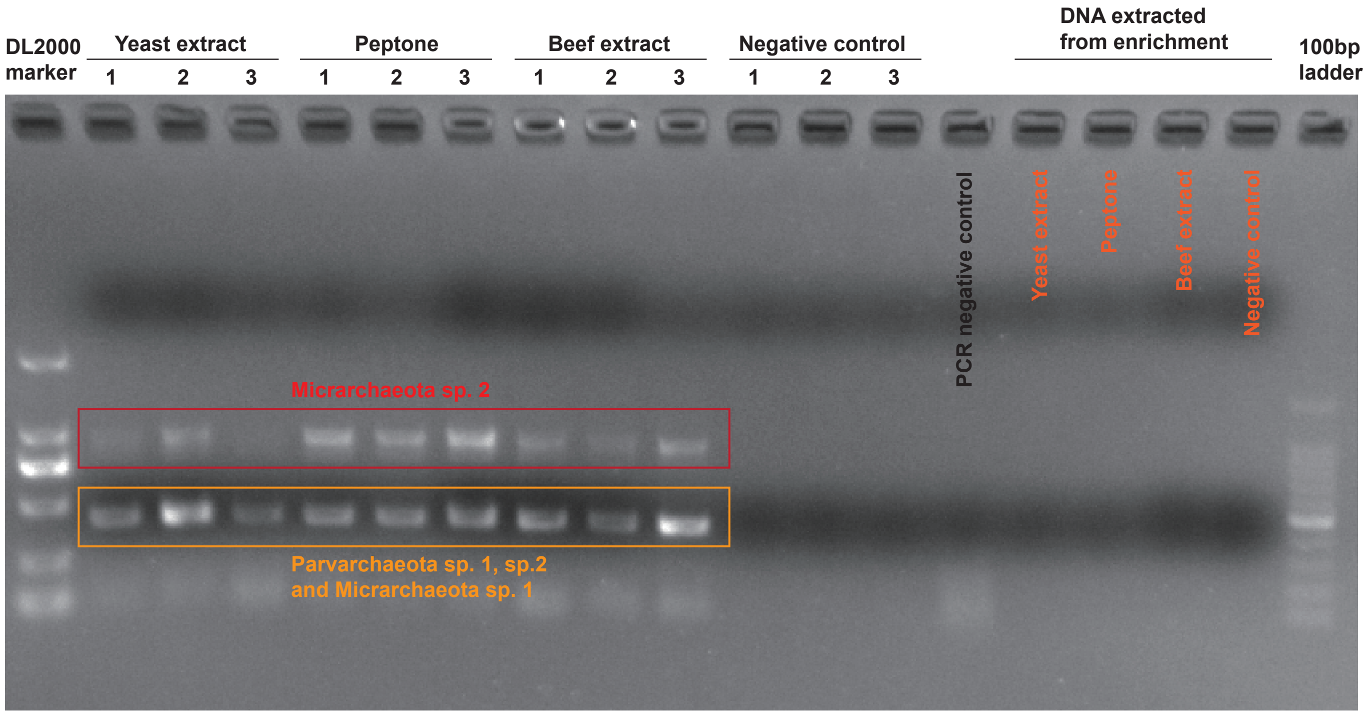

Supplementary Figure 4. Genomic DNA extracted from ARMAN enrichments (three with nutriment added and one negative control without nutriment addition) and the PCR products amplified using ARMAN specific primer set (ARM979F 5'-TATTACCAGAAGCGACGGC-3' and ARM1365R 5'-AGGGACGTATTCACCGCTCG-3'). This primer will obtain the 16S rRNA gene fragment with a length ~390 bp, but Micrarchaeota spp. usually have an insertion in their 16S rRNA gene (only Micrarchaeota sp. 2 here), thus as shown in the Figure, the gel bands in the orange box should be Parvarchaeota sp. 1 and sp.2 and Micrarchaeota sp. 1) and those in the red box should be Micrarchaeota sp. 2.

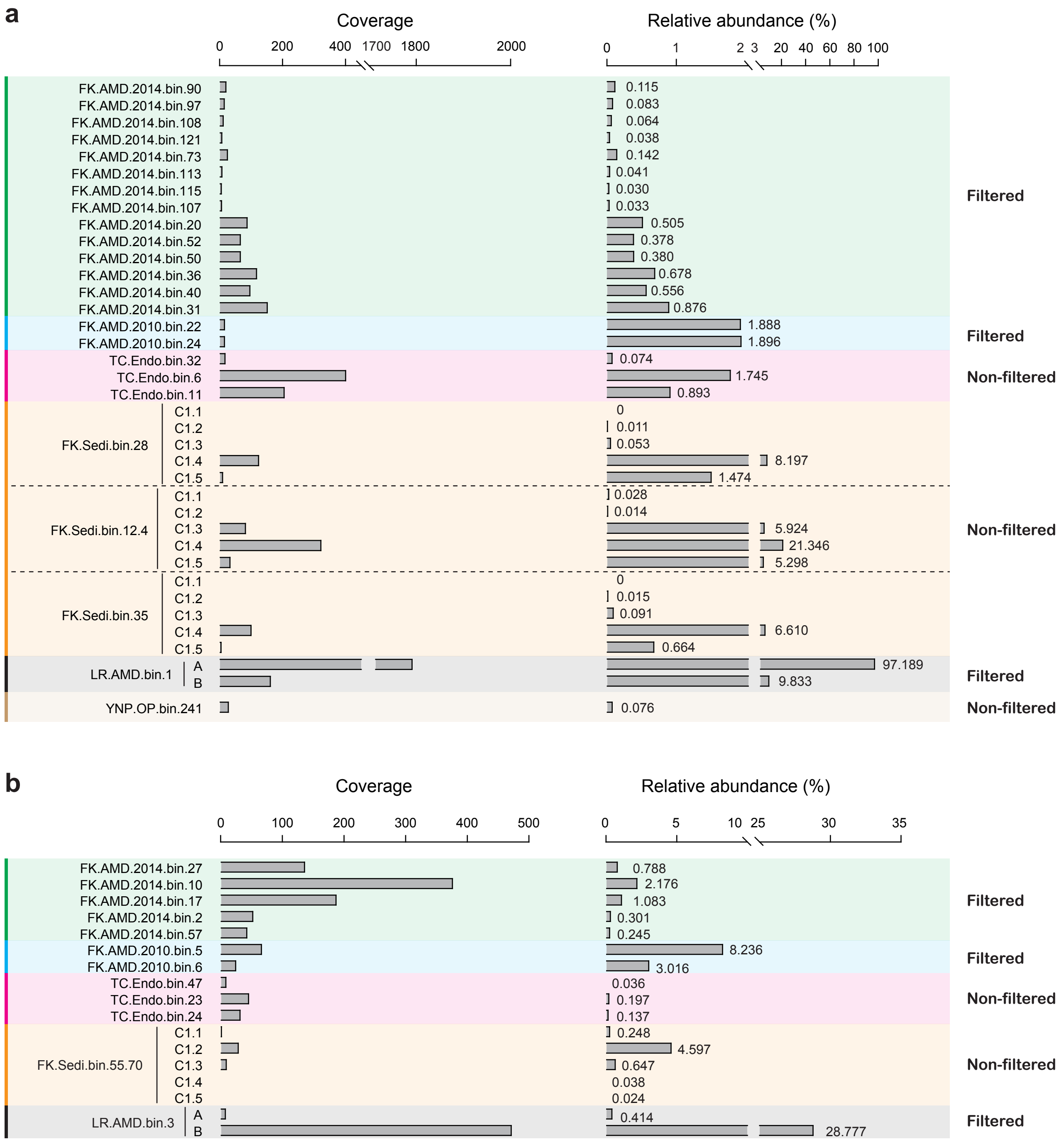

Supplementary Figure 5. The sequencing coverage of genomes in each metagenomic datasets and the relative abundance of associated taxa in each community. The ribosomal protein (RP) S3 is used for taxonomic assignment and coverage and relative abundance calculation (see Materials and methods in the main text for details). The filtering in sampling could enrich the relative abundance of ARMAN associated taxa, this was stated on the right by “filtered” or “non-filtered”.

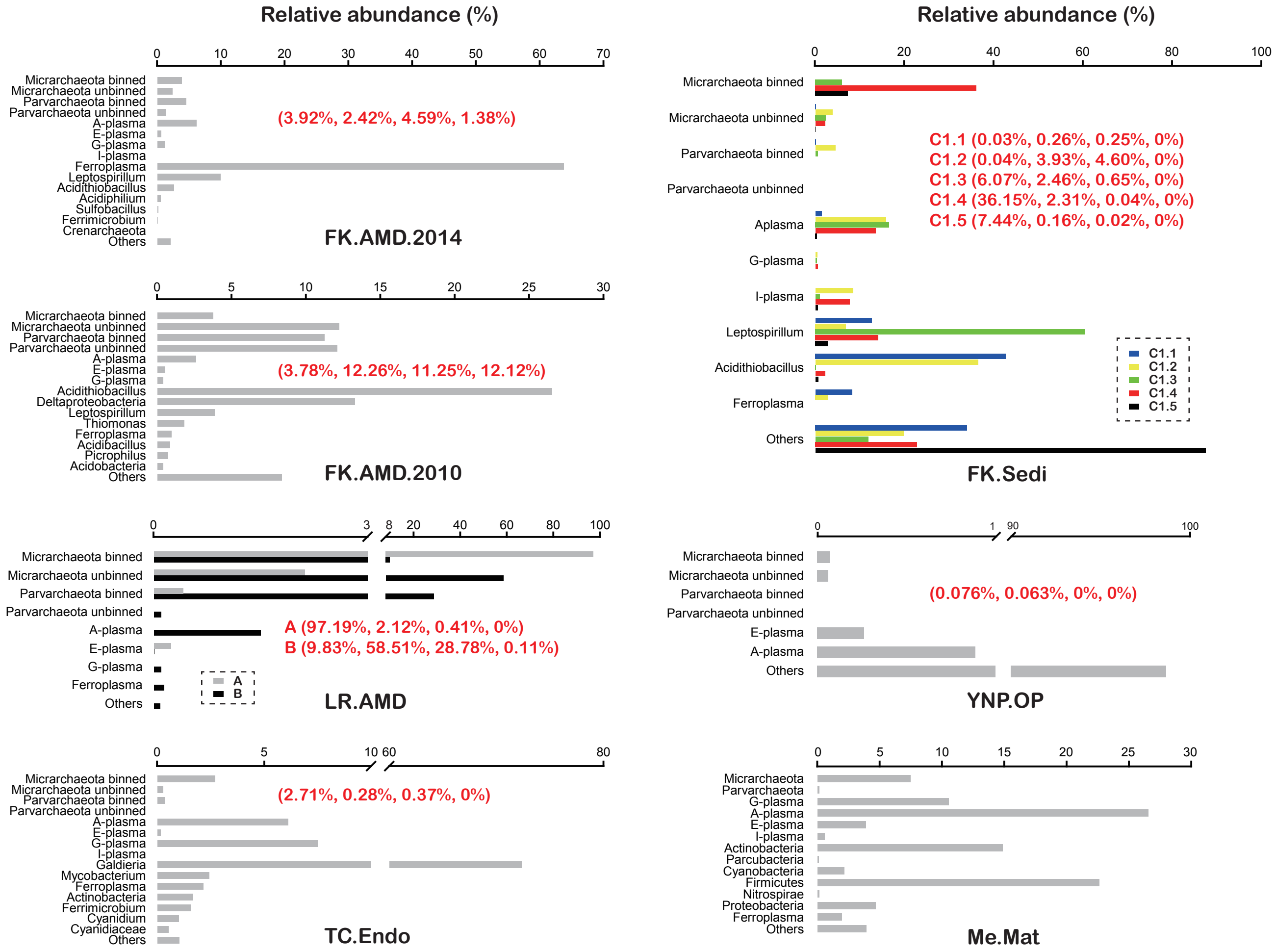

Supplementary Figure 6. The microbial composition of each community, based on the sequence coverage of the single cope marker gene of ribosomal protein S3. For both the binned and unbinned Micrarchaeota and Parvarchaeota genomes, their accumulated relative abundance were shown in the brackets (in this order: binned Micrarchaeota, unbinned Micrarchaeota, binned Parvarchaeota, unbinned Parvarchaeota). The relative abundance of alphabet plasmas were also shown, and also those dominated the communities. For FK_Sedi, “Others” were mostly sulfate-reducing bacteria, and for YNP_OP, “Others” were mostly uncultured Bacteria and Archaea.

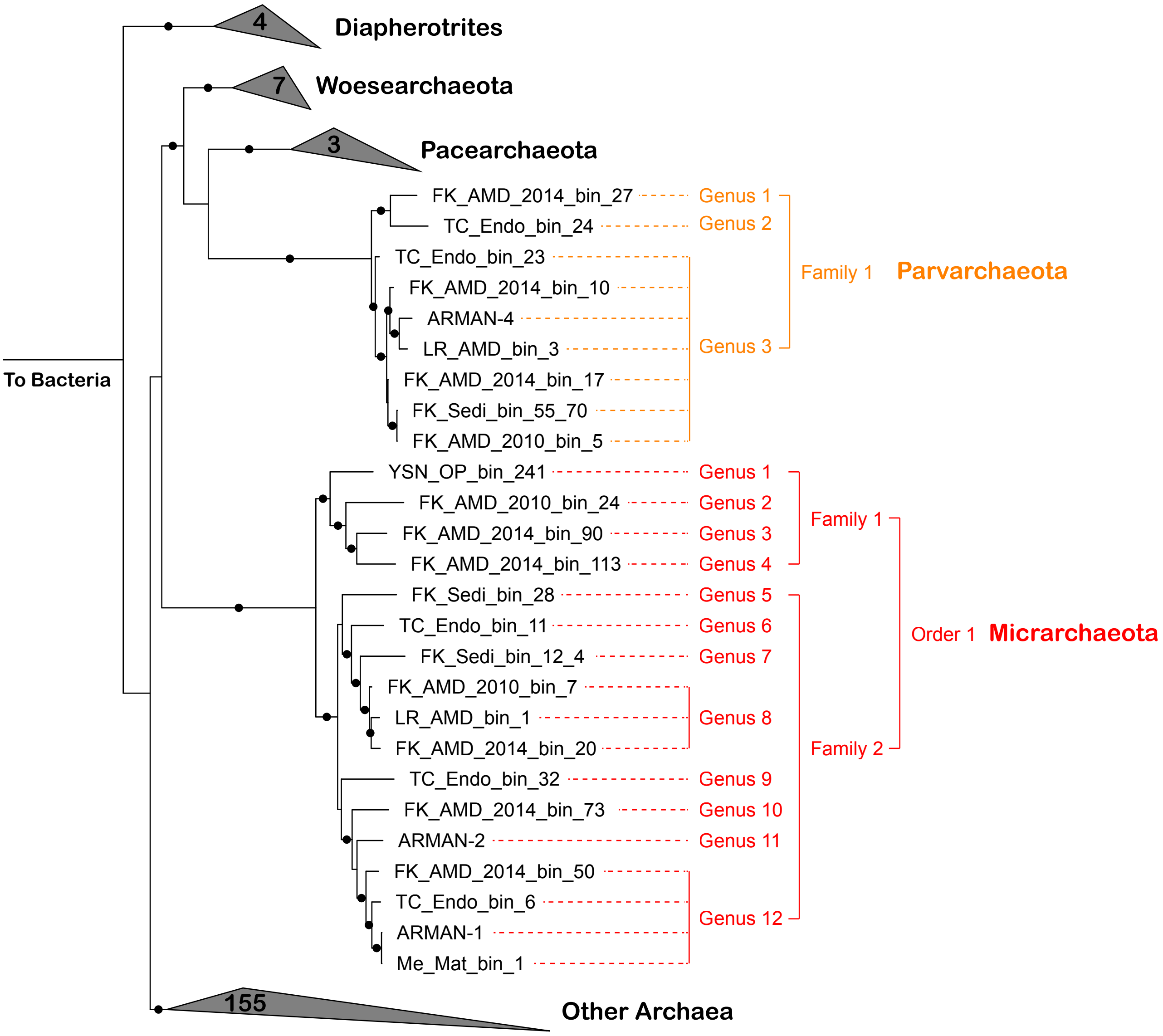

Tree scale 0.1

• Bootstrap ≥ 50%

	Genus	Family	Order	Class
Number of taxa	568	201	85	39
Median sequence identity	96.4% (96.2, 96.55)	92.25% (91.65, 92.9)	89.2% (88.25, 90.1)	86.35% (84.7, 87.95)
Minimum sequence identity	94.8% (94.55, 95.05)	87.65% (86.8, 88.4)	83.55% (82.25, 84.8)	80.38% (78.55, 82.5)
Threshold sequence identity	94.5%	86.5%	82.0%	78.5%

Supplementary Figure 7. Phylogeny and biodiversity of all ARMAN genomes based on 16S rRNA gene sequences. Only the full length 16S rRNA gene sequences include those from 17 Micrarchaeota and 9 Parvarchaeota genomes analyzed in this study, and those of other archaea. For those with insertions in 16S rRNA gene sequences, insertions (≥ 10 bp) were removed before phylogenetic analyses. The 16S rRNA gene sequences were assigned to different taxonomic levels based on sequence similarity, the thresholds from Yarza et al., 2014 were shown at the bottom. Bootstrap values are based on 100 replicates, and percentages are shown with filled circles (≥ 50%).

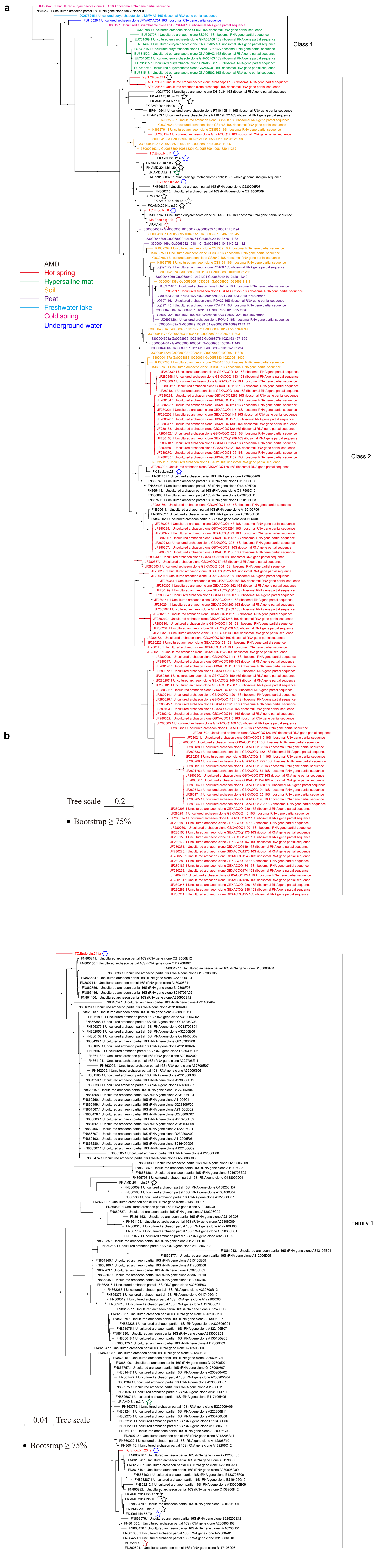

Supplementary Figure 8. The distribution of (a) Micrarchaeota and (b) Parvarchaeota in environments. All the retrieved 16S rRNA genes (≥ 800 bp) listed in Supplementary Table 7 and the full length 16S rRNA from the ARMAN genomes reported in this study were used for phylogenetical tree construction. The 16S rRNA gene sequences from different environments are indicated with different color lines, and those with genomes reported in this study are indicated with symbols as used in Figure 1 of the main text. All the 20 soil sequences were from metatranscriptomic datasets. Bootstrap values are based on 100 replicates and closed circles represent ≥ 75% bootstraps.

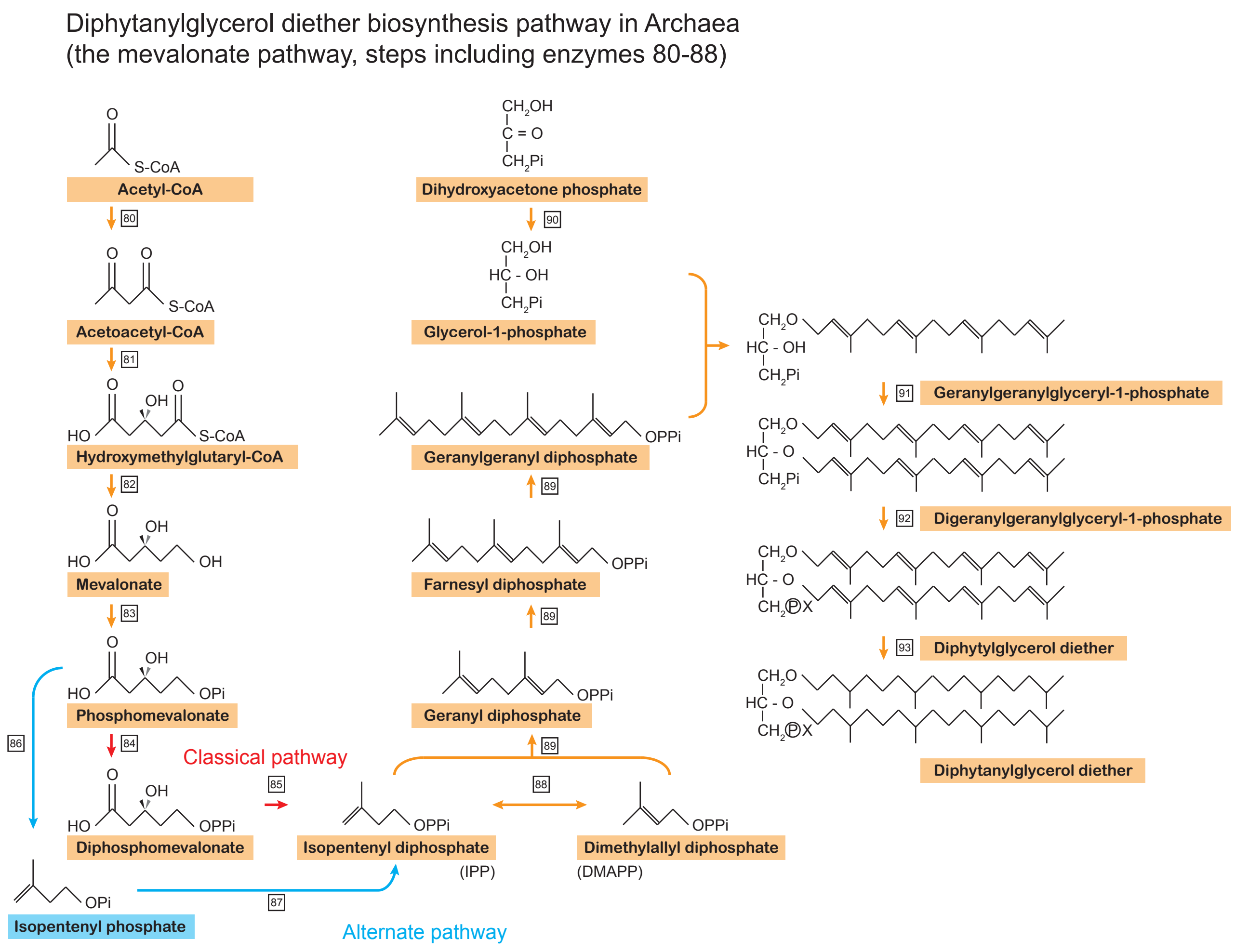

Supplementary Figure 9. Isoprenoid biosynthesis pathway in archaea. The mevalonate pathway and associated enzymes, as evidenced in archaea. The classical and alternative pathway differ in the generation of isopentenyl diphosphate, the classical pathway (as indicated in red) synthesizes it via diphosphomevalonate, while the alternative pathway (as indicated in blue) synthesizes it via isopentenyl phosphate. See Supplementary Table 8 for gene count of each enzyme.

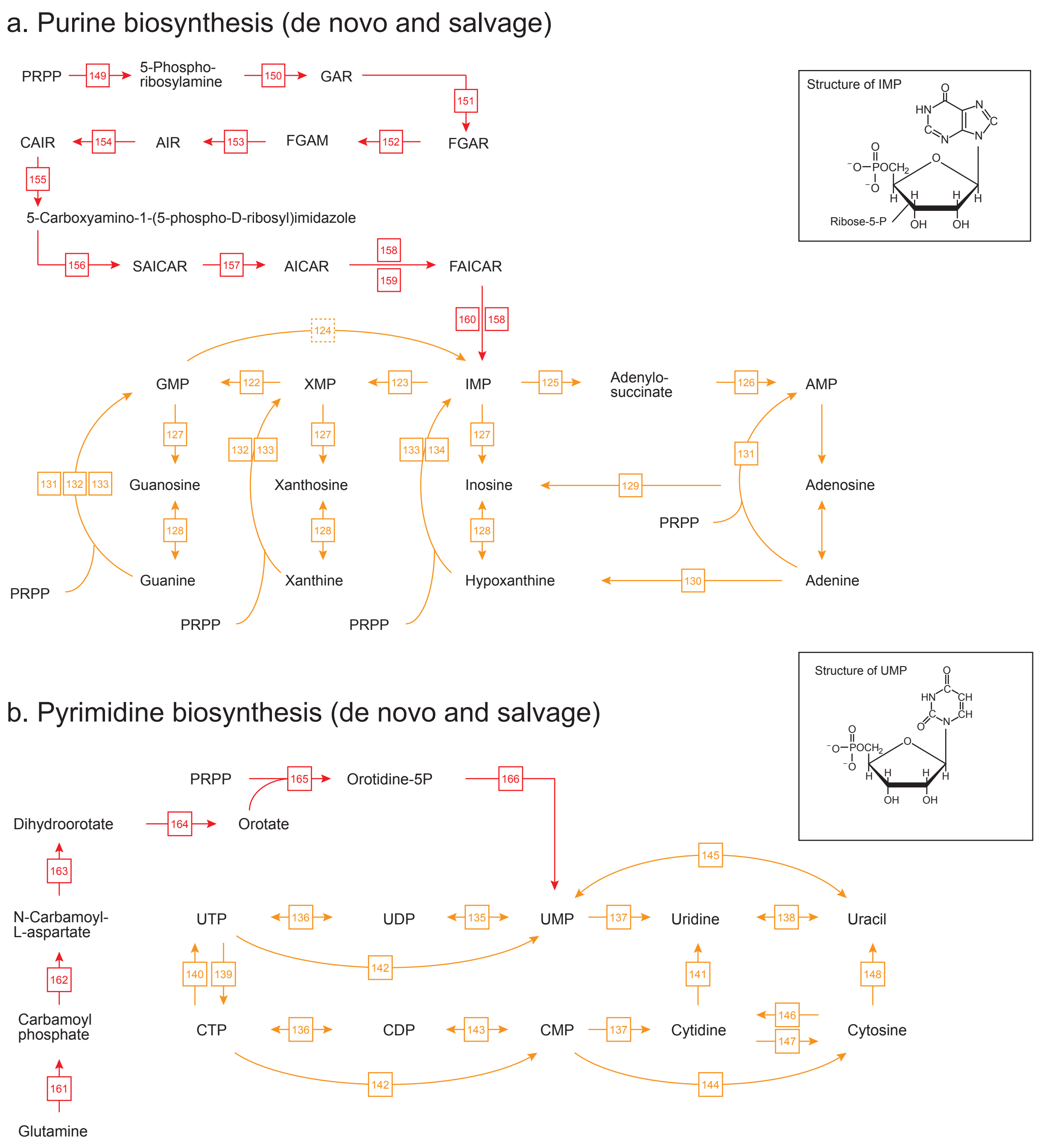

Supplementary Figure 10. The de novo biosynthesis and salvage pathway of (a) purine and (b) pyrimidine. Those indicated in red are de novo biosynthesis pathway, and those indicated in yellow are salvage pathway. The structure of the first key purine, i.e., inosinic acid (IMP), and the first key uridylate (UMP), are shown. See Supplementary Table 8 for the corresponding enzyme and gene number.

Enriched community	Quality data	Co-assembly results	Mapped reads to scaffolds
Yeast extract	5.00 Gbp	Total scaffolds = 100582;<br>Total length = 64.2 Mbp;<br>N50 = 996 bp;<br>No. of scaffolds (> 5000 bp) = 1149;<br>Longest = 896940 bp.	80.2%
Peptone	5.05 Gbp		77.4%
Beef extract	4.68 Gbp		80.5%

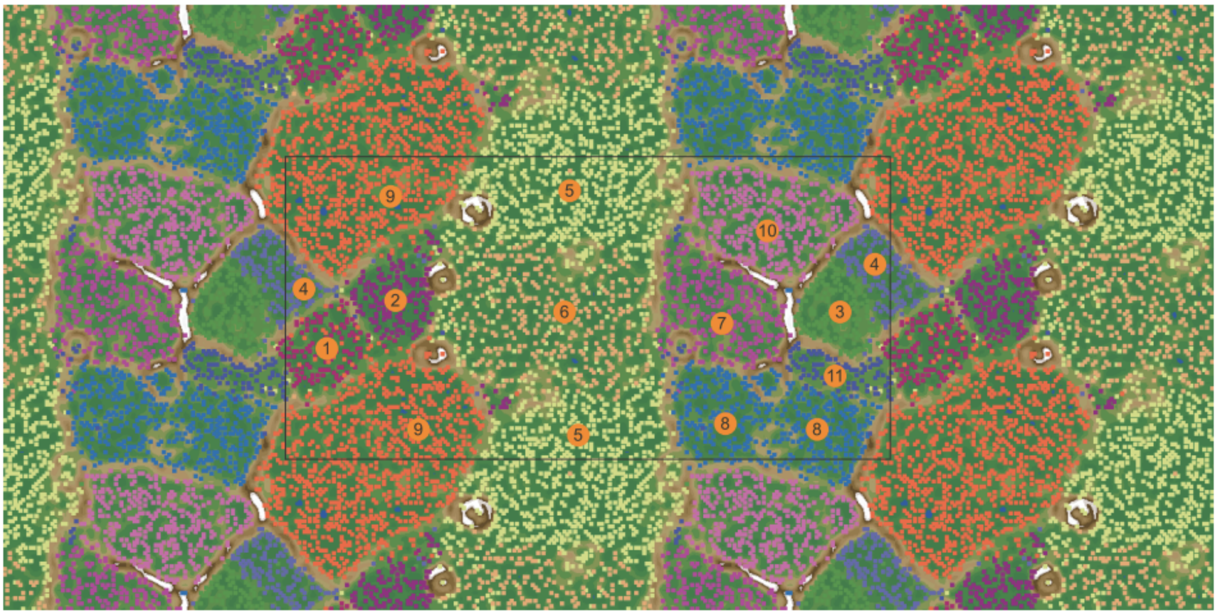

1 Parvarchaeota bin 1
2 Parvarchaeota bin 2
3 Micrarchaeota bin 1
4 Micrarchaeota bin 2
5 *Ferroplasma* related (4)
6 G-plasma related
7 *Leptospirillum* related (2)
8 *Sulfobacillus* related
9 *Pelobacter* related (2)
10 *Ferrimicrobium* related
11 Unknown

Supplementary Figure 11. Metagenomic information and ESOM based analyses of the genome bins reconstructed from enrichment communities. The taxonomic assignment of each genome bin based on the rpS3 are shown at the bottom. No attempts were performed to separate those bins (except ARMAN-related) with more than one genomes, and in such cases the number of genomes in those bins are indicated in the brackets.

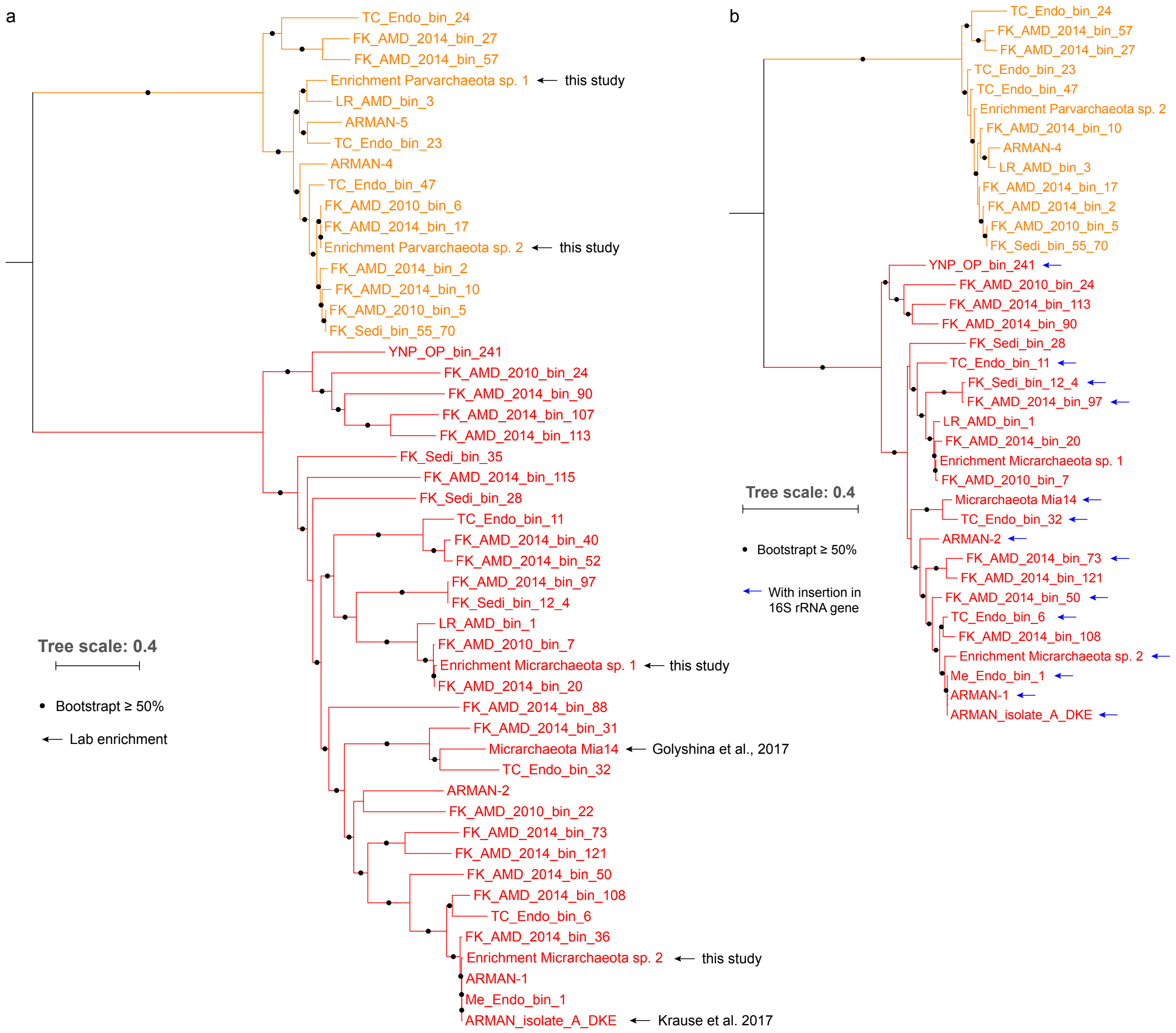

Supplementary Figure 12 Phylogenetic analyses of all the Micrarchaeota and Parvarchaeota spp. showing the phylogenesis of those from enrichment (indicated by ←). Phylogenetic analyses were based on (a) 16 ribosomal protein concatenated sequences, (b) 16s rRNA gene sequences (when available for all the ARMAN genomes, those in Supplementary Figure 7 are all full length ones).

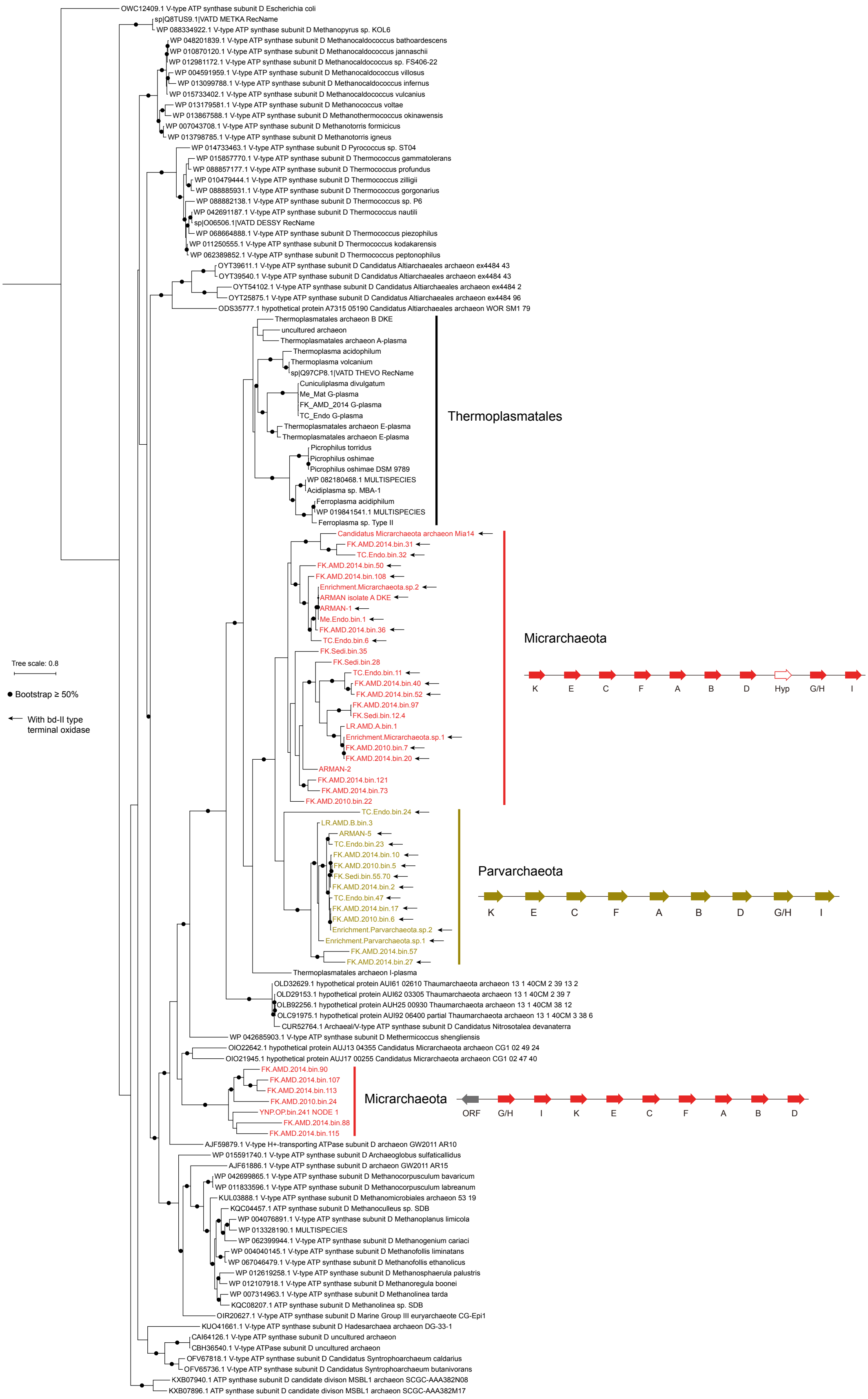

Supplementary Figure 13a Phylogeny of V-type H+-transporting ATP synthase subunit D proteins in ARMAN genomes and their homologues.

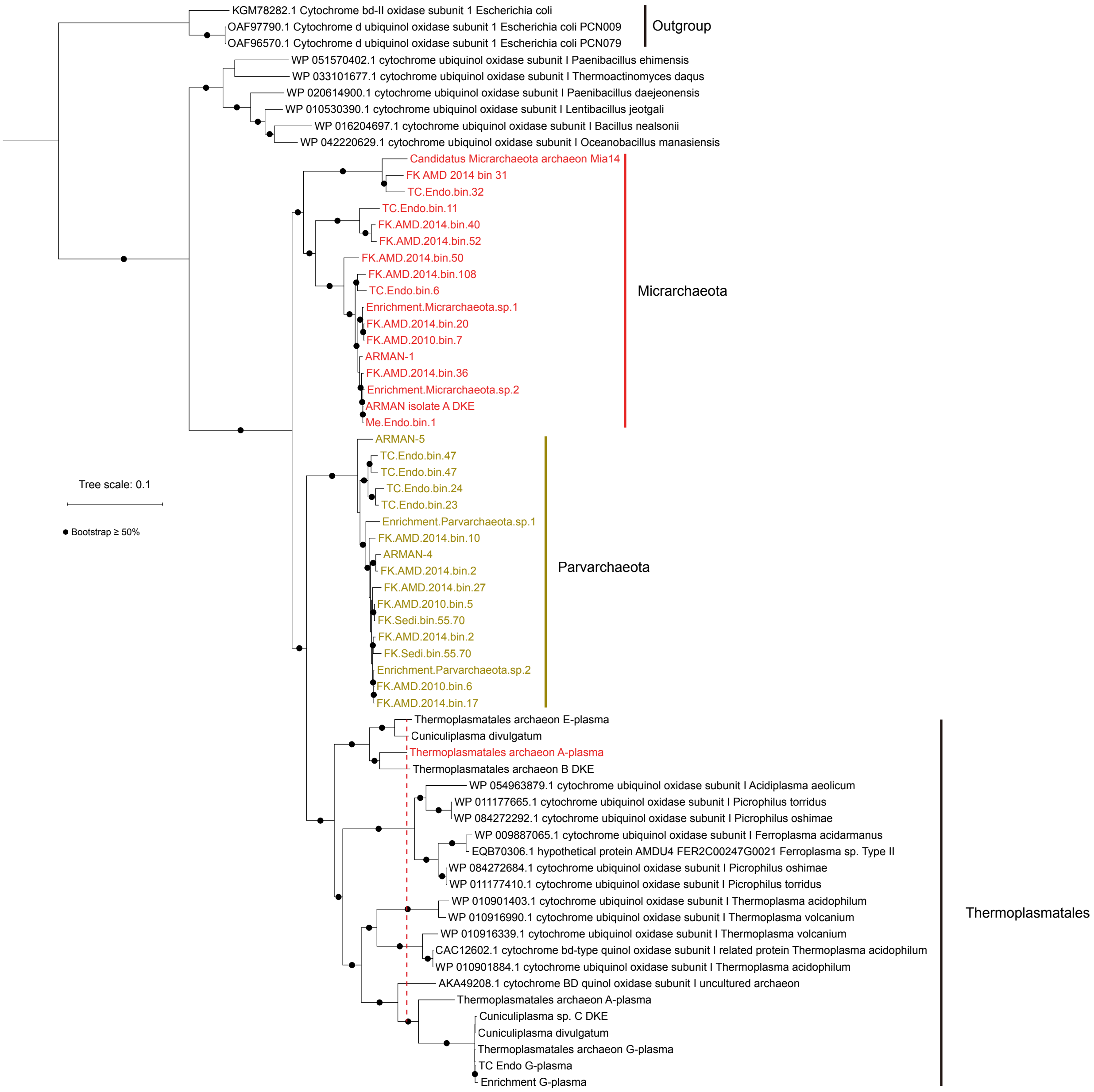

Supplementary Figure 13b Phylogeny of cytochrome bd-II terminal oxidase subunit I proteins in ARMAN genomes and their homologues.

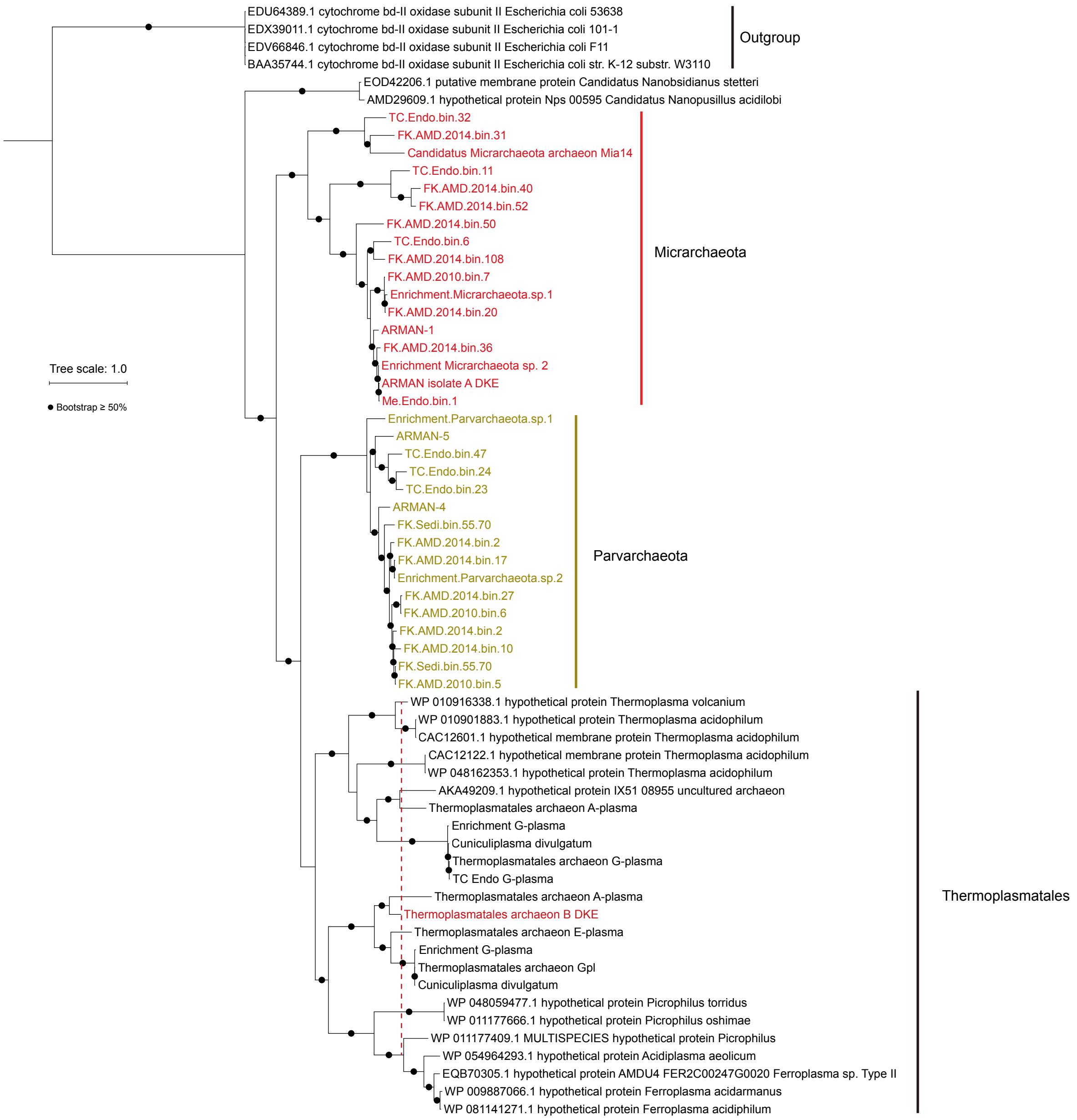

Supplementary Figure 13c Phylogeny of cytochrome bd-II terminal oxidase subunit II proteins in ARMAN genomes and their homologues.

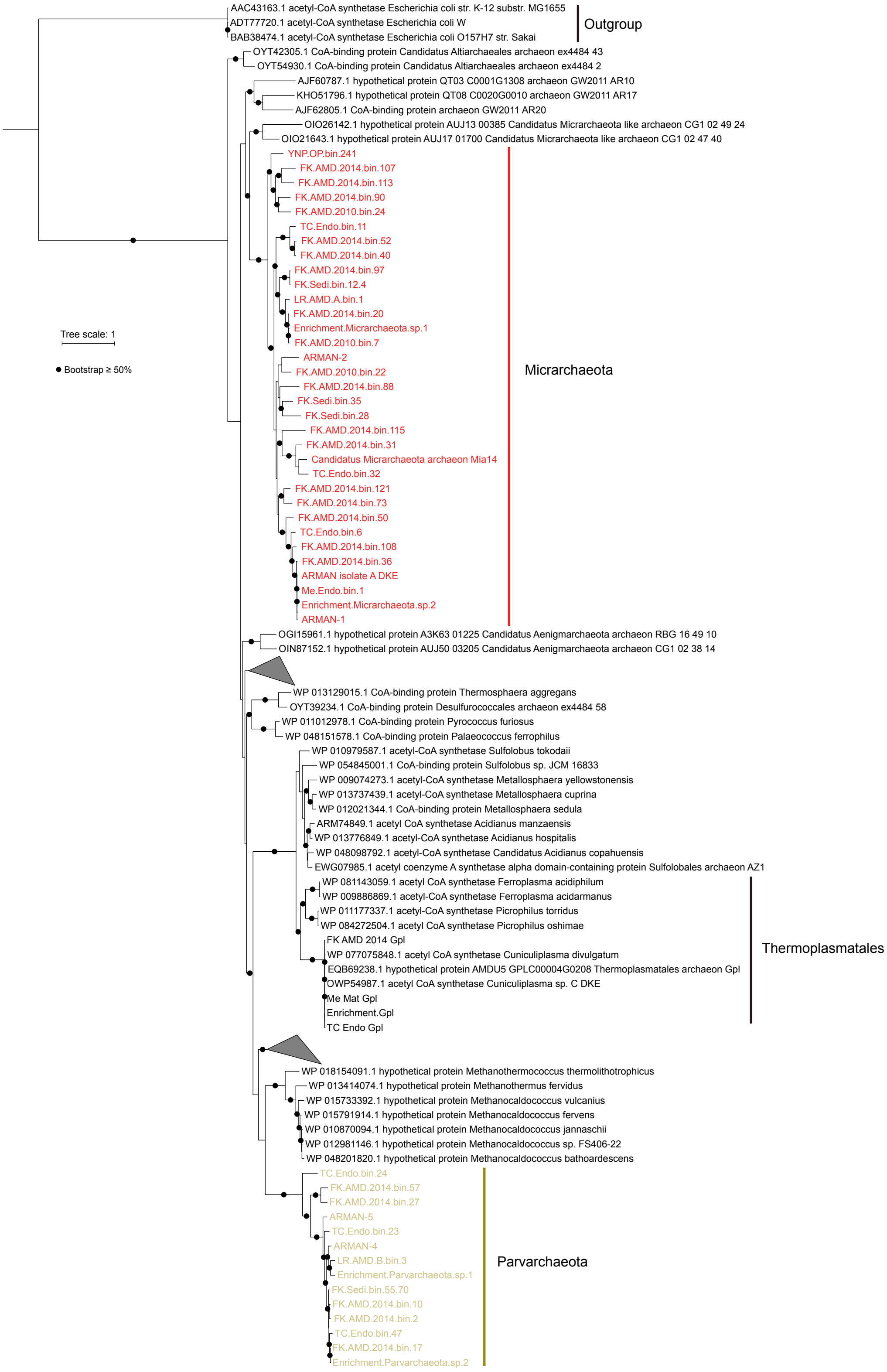

Supplementary Figure 13d Phylogeny of acetyl-CoA synthetase in ARMAN genomes and their homologues. The alpha and beta chains were concatenated before alignment.

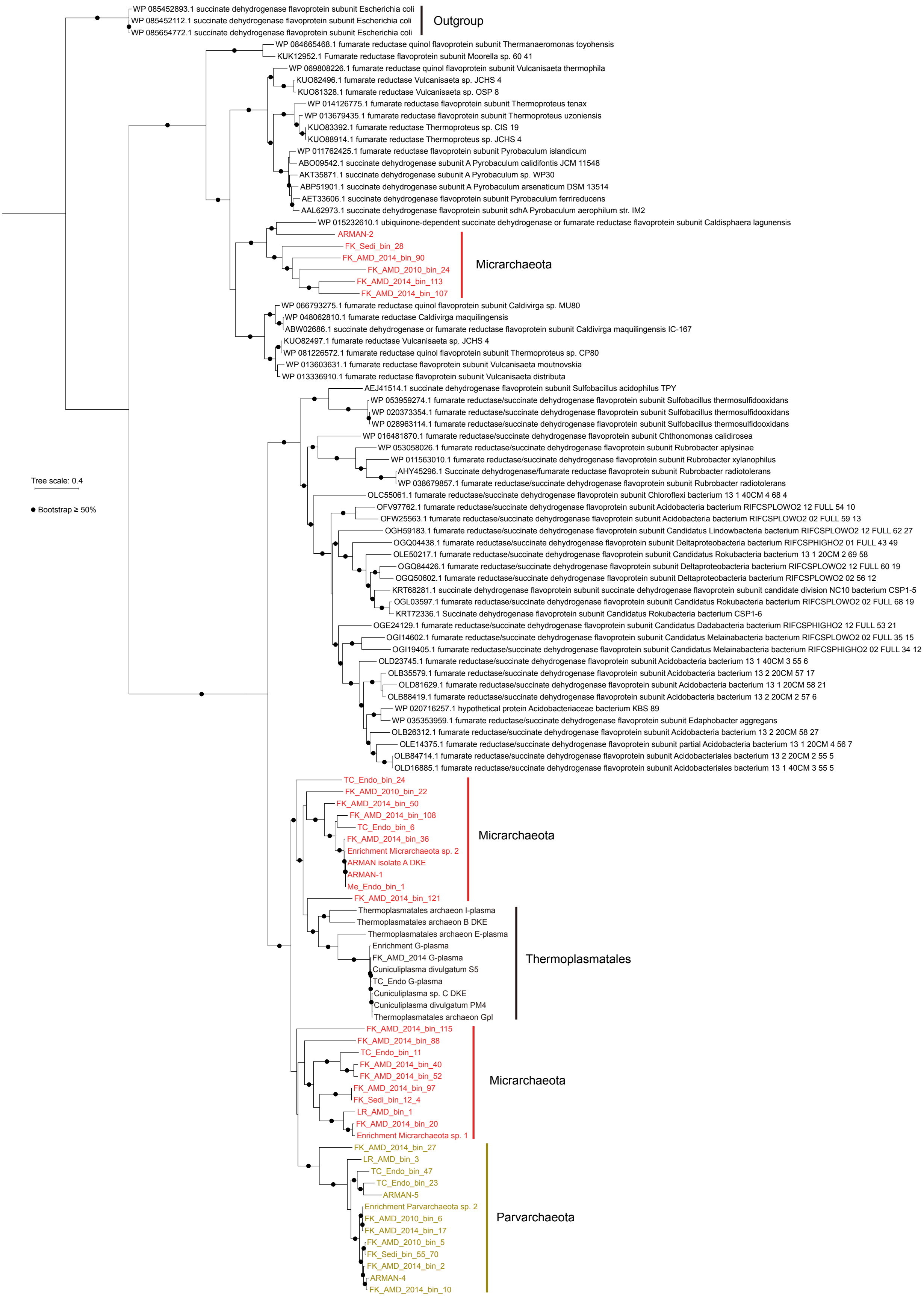

Supplementary Figure 13e Phylogeny of Succinate dehydrogenase flavoprotein in ARMAN genomes and their homologues.

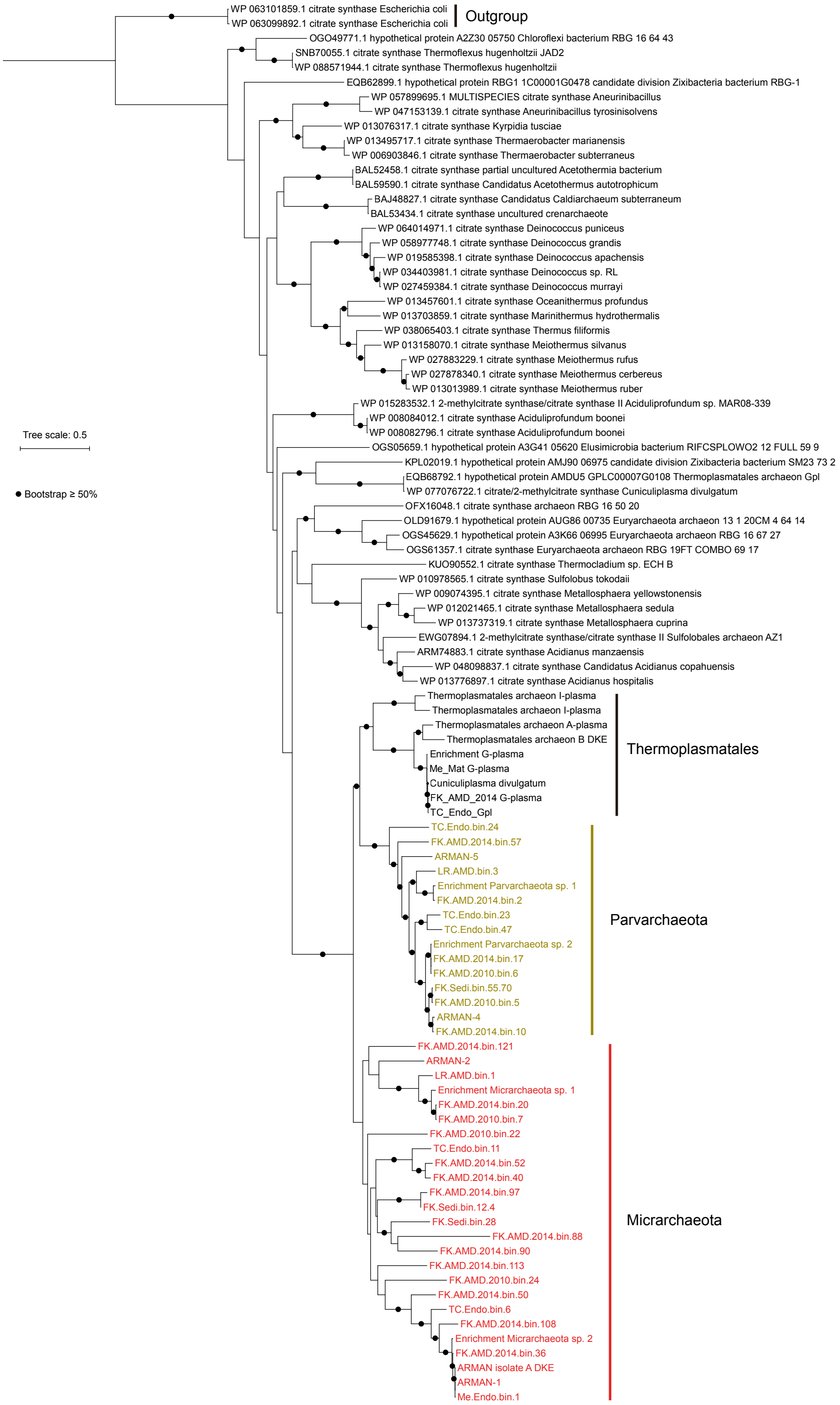

Supplementary Figure 13f Phylogeny of citrate synthase in ARMAN genomes and their homologues.

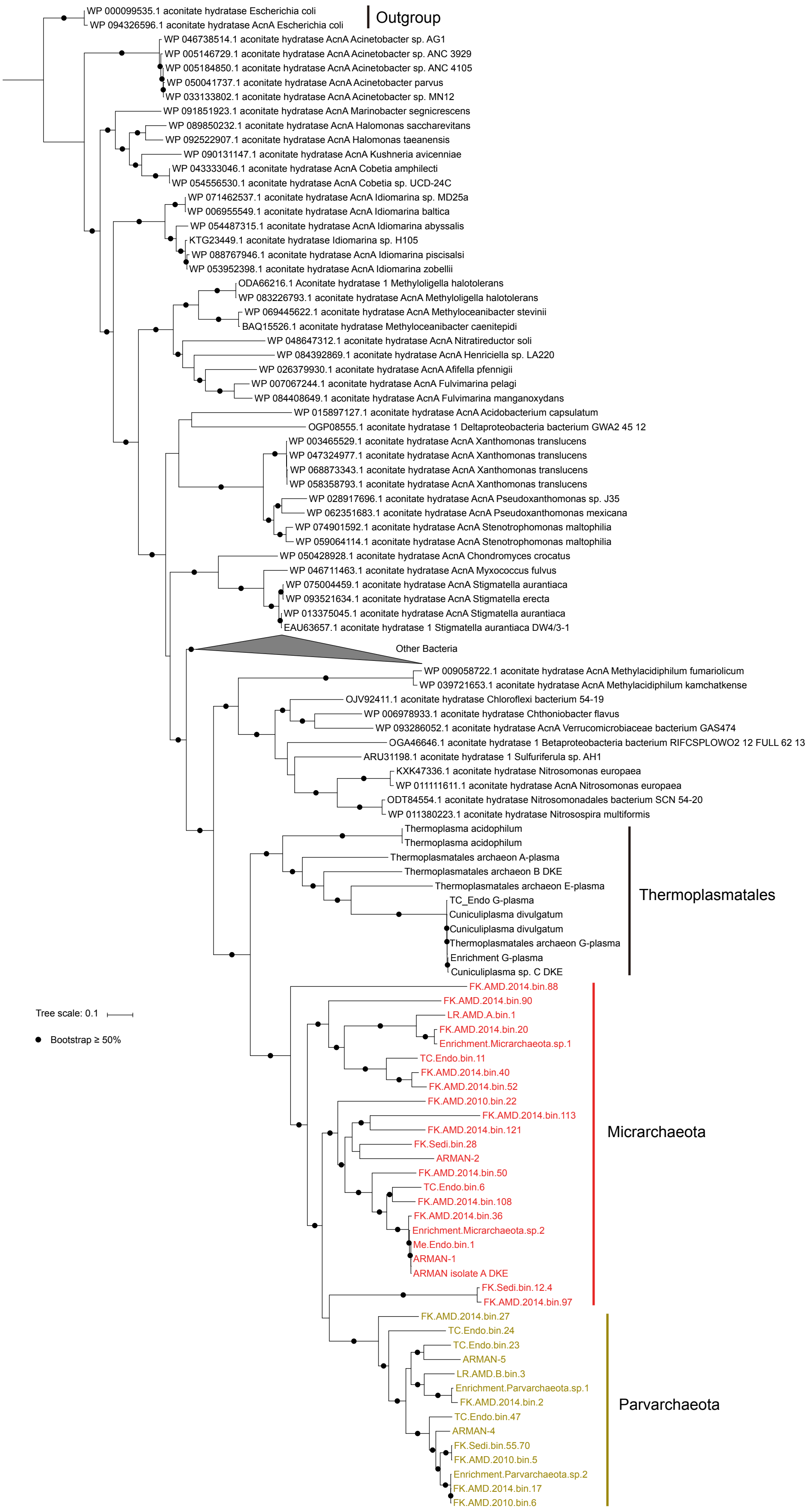

Supplementary Figure 13g Phylogeny of aconitate hydratase in ARMAN genomes and their homologues.
